# Atomically Resolved Phase Coexistence in VO_2_ Thin Films

**DOI:** 10.1021/acsnano.3c10745

**Published:** 2024-05-16

**Authors:** Masoud Ahmadi, Atul Atul, Sytze de Graaf, Ewout van der Veer, Ansgar Meise, Amir Hossein Tavabi, Marc Heggen, Rafal E. Dunin-Borkowski, Majid Ahmadi, Bart J. Kooi

**Affiliations:** † Zernike Institute for Advanced Materials, University of Groningen, Nijenborgh 4, 9747 AG Groningen, The Netherlands; ‡ Ernst Ruska-Centre for Microscopy and Spectroscopy with Electrons (ER-C), Forschungszentrum Jülich, 52425 Jülich, Germany

**Keywords:** VO_2_ thin films, transitional phases, oxygen imaging, metal−insulator transition, electron microscopy

## Abstract

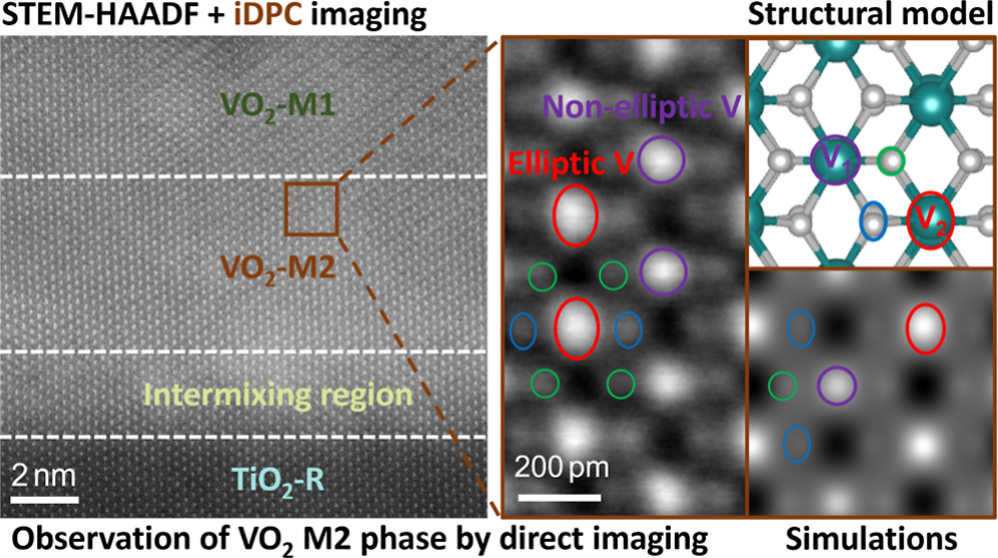

Concurrent structural and electronic transformations in VO_2_ thin films are of 2-fold importance: enabling fine-tuning of the emergent electrical properties in functional devices, yet creating an intricate interfacial domain structure of transitional phases. Despite the importance of understanding the structure of VO_2_ thin films, a detailed real-space atomic structure analysis in which the oxygen atomic columns are also resolved is lacking. Moreover, intermediate atomic structures have remained elusive due to the lack of robust atomically resolved quantitative analysis. Here, we directly resolve both V and O atomic columns and discover the presence of intermediate monoclinic (M2) phase nanolayers (less than 2 nm thick) in epitaxially grown VO_2_ films on a TiO_2_ (001) substrate, where the dominant part of VO_2_ undergoes a transition from the tetragonal (rutile) phase to the monoclinic M1 phase. Strain analysis suggests that the presence of the M2 phase is related to local strain gradients near the TiO_2_/VO_2_ interface. We unfold the crucial role of imaging the spatial configurations of the oxygen anions (in addition to V cations) by utilizing atomic-resolution electron microscopy. Our approach can be used to unravel the structural transitions in a wide range of correlated oxides, offering substantial implications for, e.g., optoelectronics and ferroelectrics.

## Introduction

The versatility and exceptional properties of VO_2_ thin films have enabled the emergence of tunable functional properties for modern switching devices, neuromorphic sensors, and optoelectronics.^1−4^ This is predominantly due to the reversible metal to insulator transition (MIT) in this correlated oxide, which occurs slightly above room temperature (∼68 °C) from metallic rutile structure (at higher temperature) to monoclinic insulator phases (at lower temperature).^5,6^ Nevertheless, the exact mechanism of the MIT with concomitant structural and electrical transformations in this scheme has remained debatable so far. From the structural point of view, an ambiguity has arisen, not only because of the complex interfacial domains but also from the lack of atomically resolved quantitative information by means of direct characterization techniques.

The MIT scheme of VO_2_ in thin films, nanowires, and bulk formats has been a subject of a long-lasting study with different aspects of attention.^7−23^ One of the less-understood features in these films are the structural alterations at the atomic scale that occur during the transition. These films undergo a martensitic-like first-order transition from a high-temperature tetragonal metallic phase, i.e., rutile R phase with space group: *P*4_2_/*mnm*, to a low-temperature insulating monoclinic phase, i.e., M1 with space group: *P*2_1_*C*.^24^ In addition to these typical phases, some metastable and transitional phases including M2, T, and xM3 have been also reported.^12,25−28^ Mediated by strain, a complex phase coexistence in these films has been identified.^29^ Among the phases present, the monoclinic M2 (space group: *C*2/*m*) has received particular attention as it provides extensive opportunities (superior to M1) for electronic property modulation, memory applications, and thermal actuators.^30,31^ Nonetheless, the spatial foundation and atomic structure characteristics of the M2 phase and its relation to MIT require further understanding.

So far, the transitional domains during the MIT in VO_2_ have remained unresolved at the atomic scale due to their complexity. Intermediate phases (specially the M2 polymorph) have been either predicted theoretically using, e.g., density functional theory (DFT) calculations^32^ or spotted by Raman spectroscopy,^33^ X-ray diffraction,^29^ and conductive atomic force microscopy.^34^ However, these methods offer characteristic information on a more global scale and lack local atomic-scale detection of these phases with exact spatial coordination (distribution), particularly in thin films. Naturally, atomic-scale characterization would provide invaluable information on the MIT, as it initiates at this scale and can enable tunable, tailored-made functional materials and switching devices. Imaging the oxygen atoms (V–O bonds) in these films with aberration-corrected scanning transmission electron microscopy (STEM) can reveal atomic-scale electrical or structural changes enabled by oxygen atom configuration or displacement. In this context, the approach presented here can be used for quantifying the local atomic structures in a wide range of correlated oxides.

## Results and Discussion

**Figure 1:**
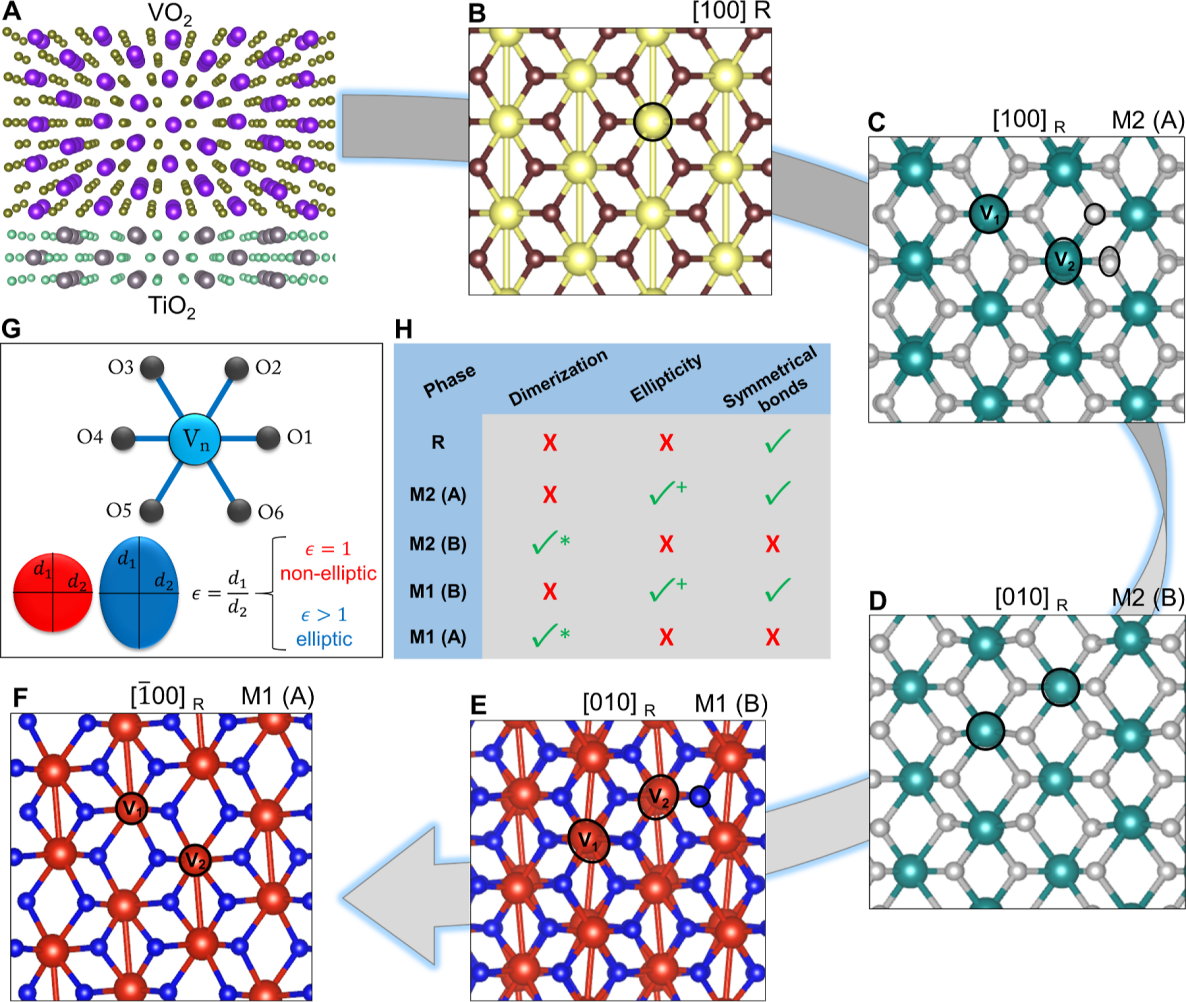
Crystallographic representations of observed structures in the MIT scheme and the criteria to distinguish these structures. The crystallographic representations of the (A) epitaxially grown VO_2_ on a TiO_2_ (001) substrate, (B) TiO_2_ rutile (R) phase in [100]_R_ projection, (C) intermediate insulating monoclinic M2 (A) phase in [100]_R_ projection, (D) intermediate insulating monoclinic M2 (B) phase in [010]_R_ projection, (E) insulating monoclinic M1 (B) phase in [010]_R_ crystal projection, (F) insulating monoclinic M1 (A) phase in [1®00]_R_ projection, (G) archetypal VO_6_ octahedron indicating the labeled atoms together with the ellipticity concept, and (H) incorporated criteria to differentiate the present phases. + Note that M2 (A) and M1 (B) both generate clear ellipticity in their projections, yet the ellipticity of V atoms in M2 (A) only occurs every second atomic column (i.e., in V2), whereas the ellipticity of M1 (B) occurs for all the V atomic columns. * Notice that the dimerization of (edge-on observed) V–V planes in M1 (A) is more pronounced than that of M2 (B).

The basis of our work is the successful growth of high-quality VO_2_ thin films on TiO_2_ (001) substrates using pulsed laser deposition (PLD) (Figure S1), schematically shown in Figure 1A. Only for this substrate and specific surface orientation, the VO_2_ atomic structure can be unambiguously resolved in projected images, for which we also provide more background information in another paper.^35^ We start here by schematically revealing the different structures in the specifically chosen crystallographic projections that play a key role in our approach to properly resolve all of the atomic structure details in our VO_2_ films. An overview scanning electron microscopy image of the VO_2_ domains is shown in Figure S2. As Figure 1 depicts, the VO_2_ grown at a temperature of 400 °C undergoes a martensitic-like transformation (at about 68 °C) from the rutile R phase (panel B) to the intermediate phase M2 (panels C and D) reaching the M1 phase (panels E and F) at room temperature. The VO_2_ film is epitaxially grown on the (001) surface of TiO_2_. Then, when the VO_2_ is imaged in cross sections with the interface edge-on, the M2 and M1 phases will be observed along two orthogonal crystallographic projection directions because for the rutile (tetragonal) structure, [100] and [010] are equivalent, but then for the monoclinic phases (M2 and M1), two nonequivalent viewing directions will occur, which we refer to as A and B directions. The M2 (A) type refers to the projection along TiO_2_ [100]_R_ and M2 (B) along TiO_2_ [010]_R_ as depicted in Figure 1C,D, respectively. M1 (B) and M1 (A) denote the projections along [010]_R_ and [1®00]_R_, respectively. In Figure 1G,H, we introduce our criteria to distinguish the different phases. In Figure 1G, the archetypal VO_6_ octahedron for both the TiO_2_ (VO_2_) tetragonal R phase and monoclinic M phases is given. Note that apart from a small lattice parameter change, the VO_6_ octahedron is identical for the R phases of TiO_2_ and VO_2_ (where the latter is only stable above about 68 °C). The vanadium atom is positioned in the center of the octahedron surrounded by six oxygen atoms labeled from O1 to O6, where each O atom possesses a specific distance from the central V (i.e., V–O bond distance) and a certain angle in different configurations during the MIT. Table S1 provides the calculated theoretical values of bond distances and angles for the R and M structures. We also introduce the concept of ellipticity of the atoms in Figure 1G, indicating the aspect ratio of the projected atoms in a column on an imaging zone axis. The ellipticity shows not only a size but also a directionality, which is a useful criterion to be verified experimentally. Figure 1H then shows a table where the different criteria, including atomic ellipticity, symmetry of V–O bonds with respect to the central V atom, and dimerization of the (edge-on observed) V–V planes, are applied to differentiate the structures in their different projection directions. Dimerization is defined as the alternating shorter and longer bond distances between V atoms (Peierls-like distortion), which lead to alternating shorter and longer distances between V atomic planes. For our STEM analysis, it is then important to have these planes observed edge-on, which is achieved for the epitaxy and projection direction we use here. For instance, in Figure 1F for the M1 (A) structure, the V–V dimers are made visible by the red bars (bonds) connecting two V atomic columns. Overall, Figure 1, in particular the table in Figure 1H, demonstrates that it is possible to distinguish the R, M2, and M1 phases in their different projections unambiguously.

**Figure 2:**
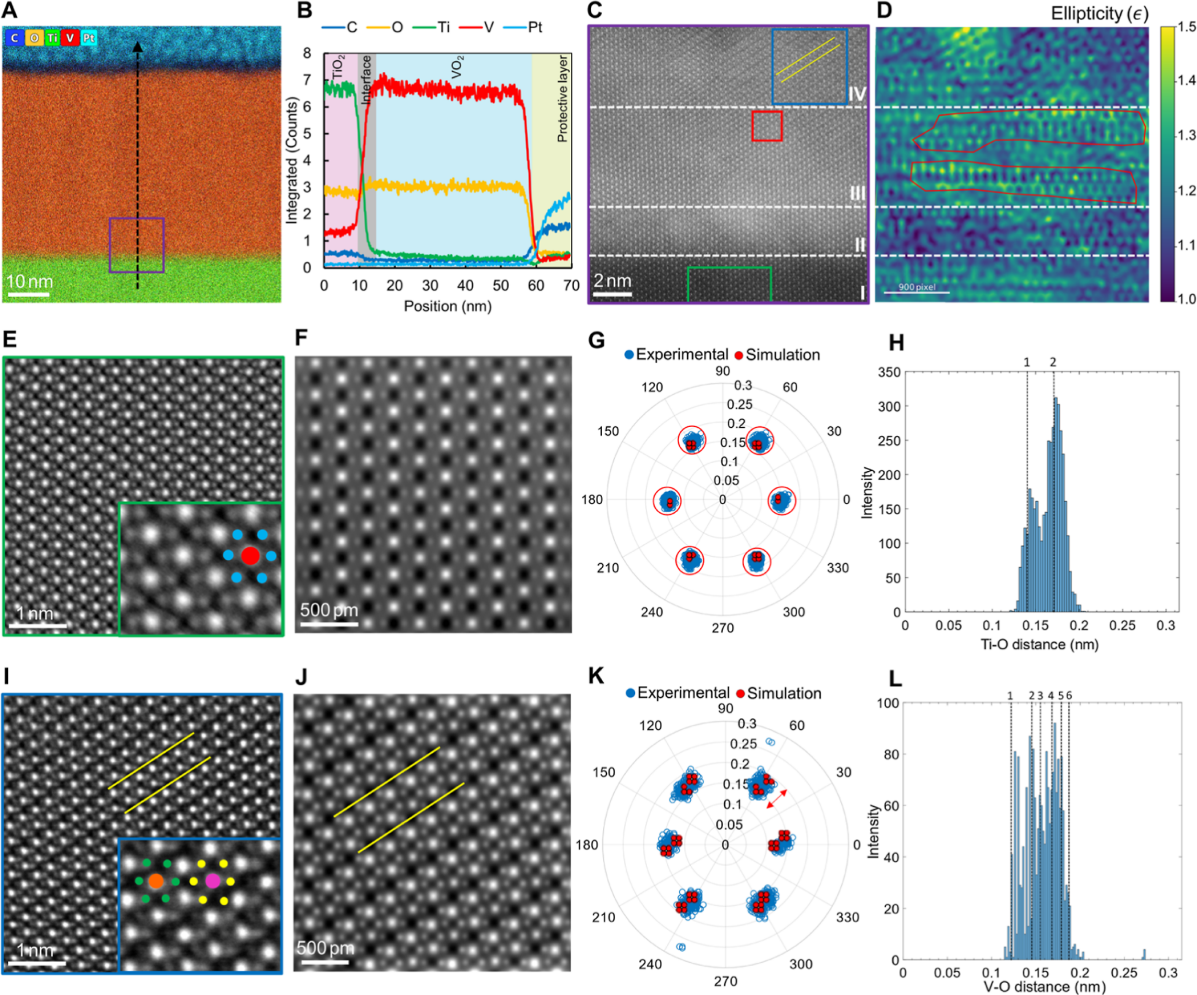
Quantitatively resolving the oxygen atoms in the rutile R and dimerized M1 phase. (A) EDS map of a cross section of the film (in the focused ion beam-cut TEM lamella), (B) EDS elemental profiles along the indicated arrow in panel (A), (C) high-angle annular dark-field (HAADF) image of the various domains of the VO_2_ film on TiO_2_, (D) atomic ellipticity analysis on the entire region, M2 phase domains are designated by red contours, (E) integrated differential phase contrast (iDPC) image of the R phase, (F) simulated iDPC micrograph of the R phase, (G) quantified spatial configurations of the oxygen atoms for R showing symmetrical bonds (the units for angles and distances are deg and nm), (H) distribution of the Ti–O bond distances compared to theoretical values shown as 1 and 2 dashed lines, (I) iDPC image of the dimerized M1 (A) phase, (J) simulated iDPC image of the dimerized M1, (K) quantified spatial configurations of the oxygen atoms for M1 (A) revealing nonsymmetrical bonds, and (L) distribution of the V–O bond distances compared to theoretical values indicated as 1–6 dashed lines (12 measured bonds are grouped into 6 pairs as described in the Supporting Information).

We continue by showing high-resolution STEM results of the studied VO_2_ thin film. Figure 2 depicts experimental STEM images of a cross section of the film, the corresponding simulated STEM images, and postprocessing quantification of atomic structures in both experimental and simulated images. A detailed description of the focused ion beam lamella preparation and STEM imaging is provided in the Supporting Information. In order to quantify the V–O bond coordinations and lengths in a statistical manner, we have developed a MATLAB code specifically for this purpose (see Supporting Information).

As can be observed in the EDX results in Figure 2A,B, the representative selected area in the cross section consists of the VO_2_ film on top of the TiO_2_ substrate also including the interface (with a thin intermixed region). However, the experimental atomic-resolution HAADF–STEM image in Figure 2C provides more details about the film. Four different zones are resolved, namely, TiO_2_ substrate (zone I), intermixing region (zone II), VO_2_ region containing transitional domains M2 (zone III), and the VO_2_ region fully composed of M1 (zone IV). The corresponding ellipticity map of the selected area is depicted in Figure 2D, where particularly in zone III, distinct periodic patterns can be observed.

The iDPC-STEM image of the TiO_2_ (001) substrate viewed along [100]_R_ in Figure 2E shows perfectly symmetrical Ti–O bonds, where oxygen atoms are distinctly resolved in accordance with Figure 1B. The simulated iDPC-STEM image of the same zone axis in Figure 2F confirms the revealed structure of the R phase. The quantification of the Ti–O bond distances and the spatial configurations of the oxygen atoms with the corresponding angles for the R phase are provided in Figure 2G. The simulation results show that the oxygen atoms are accurately imaged in the zone axis with a precise spatial configuration in accordance with the R phase model. We statistically confirm and measure the coordination of the O atoms, which corresponds well with the theoretical calculations given in Table S1. To further elucidate the structure, the theoretical values of Ti–O bond distances are compared (via dashed lines) to the measured experimental ones in Figure 2H. Indeed, the two short and four long Ti–O bond distances are distinctively resolved, resulting in two different peaks with the correct distances and intensity ratio of 1:2 in the histogram.

The experimental and simulated iDPC-STEM images of the VO_2_ M1 (A) phase (from zone IV) are shown in Figure 2I,J, respectively. These images clearly reveal the V–V dimerized atoms (the dimerization direction is defined by the tilted yellow lines), together with the oxygen atoms. The resolved structure is in accord with the M1 (A) crystal configuration (Figure 1F) that exhibits two types of vanadium atomic columns: V1 and V2 because one of the two has the shortest bond distance to the O atomic column toward the right and the other toward the left. Therefore, the visualization and quantification of oxygen atom positions using the iDPC images in Figure 2I–L provides insightful observations. First, one can notice that, in contrast to the R phase, the V–O bonds are not symmetrical anymore in this M1 phase. Second, the O atoms possess a certain directionality relative to the dimerization direction. Each V atomic column has its six neighboring O atomic columns at 6 different distances (and bond angles). However, these distances are inverted for neighboring V atomic columns, as highlighted in the inset of Figure 2I by the orange and pink dots on neighboring V atomic columns surrounded by their 6 O atomic columns indicated by green and yellow dots, respectively. The simulated results indeed demonstrate that there exist 12 calculated V–O bond types in the M1 (A) phase (see Figure 2K red dots and also Table S1). The plot in Figure 2L elucidates the values of the V–O bond distances derived from statistical analysis of the experimental image, in comparison with theoretical values. Note that the 12 different V–O bonds from Figure 2K and Table S1 then boil down to six distances in Figure 2L (because directionality/angles are lost and only distances remain). As can be conceived from Figure 2L, in contrast to the distinct two peaks observed for the R phase, here obviously a larger variety of V–O bond peaks occurs. Therefore, the atomic structure electron microscopy analysis clearly distinguishes the R phase and M1 (A) in accordance with the criteria that were set earlier in Figure 1H, i.e., a phase that does not exhibit dimerization and ellipticity but has symmetrical bonding is necessarily the R phase, whereas the VO_2_ phase which does not possess ellipticity and symmetrical bonds but exhibits dimerization of V–V atoms is indeed the M1 (A) polymorph.

**Figure 3:**
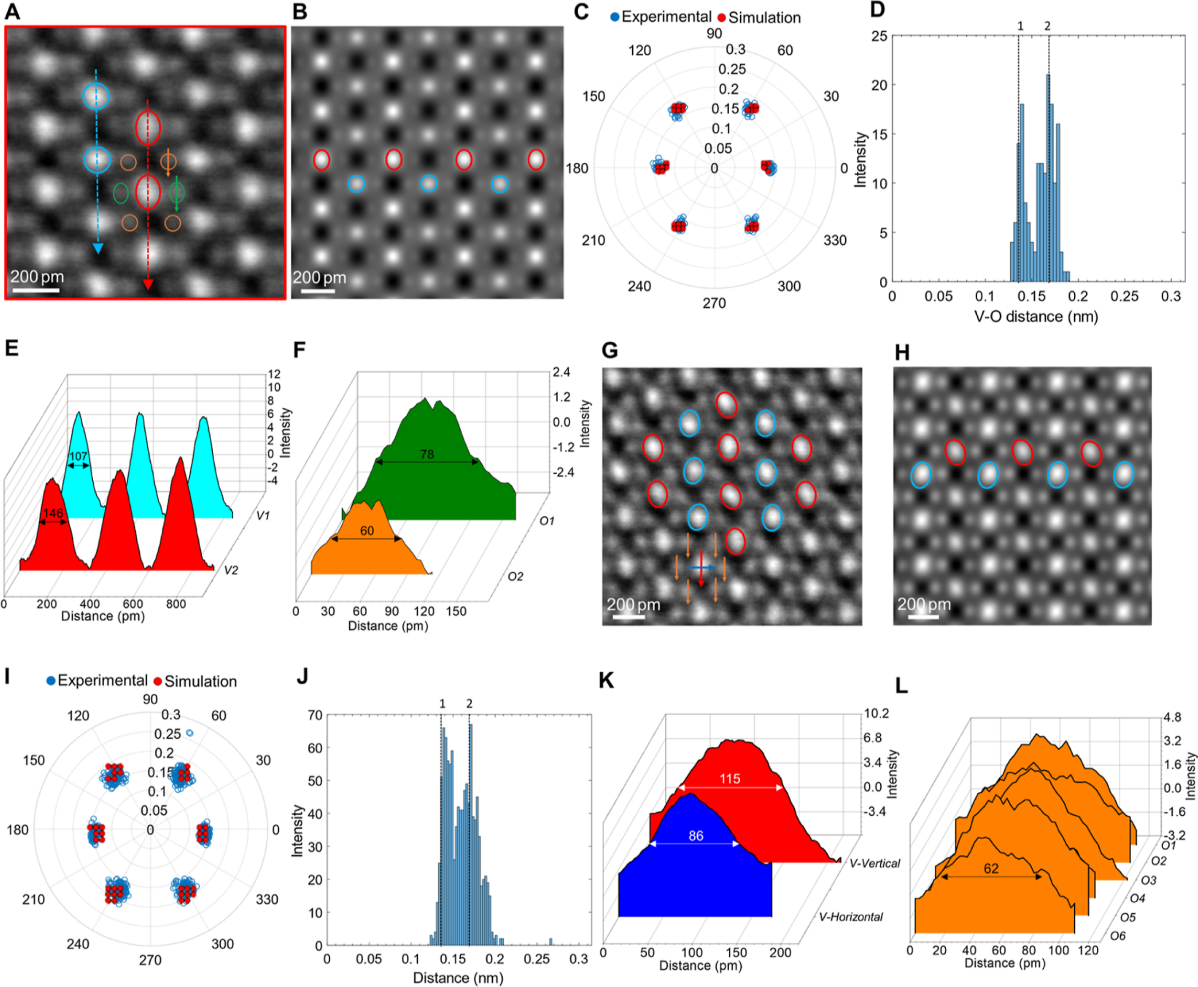
Real-space imaging of the transitional phase. (A) Experimental iDPC image of the detected intermediate M2 (A) phase, (B) simulated iDPC micrograph of the M2 (A), (C) quantified spatial configurations of the oxygen atoms for M2 (A) (the units for angles and distances are deg and nm), (D) distribution of the V–O bond distances compared to theoretical values shown as 1 and 2 dashed lines, (E) intensity profiles along the V atoms indicated in panel (A) demonstrating the alternating ellipticity in the V columns, (F) intensity profiles along the O atoms indicated in panel (A) showing ellipticity in O1, (G) iDPC image revealing the M1 (B) phase, (H) simulated iDPC image of the M1 (B) phase, (I) quantified spatial configurations of oxygen atoms for M1 (B), (J) distribution of the V–O bond distances compared to theoretical values shown as 1 and 2 dashed lines, (K) intensity profiles on the V atom indicated on panel (G) showing atomic ellipticity, and (L) intensity profiles along the O atoms designated in panel (G).

Next, we proceeded with the elaboration on the M1–M2 phase coexistence in the epitaxially grown VO_2_ thin films. As shown in Figure 2D, an inhomogeneous atomic ellipticity throughout the cross section of the film is found. Surprisingly, right in zone III, one can notice in certain regions a distinct pattern in the ellipticity map, as indicated by the red contours in Figure 2D. Although the imaging is performed completely in the zone axis for the TiO_2_ substrate below zone III and the VO_2_ M1 phase above zone III, atomic columns in a large fraction of zone III show substantial ellipticity in an alternating pattern parallel to the edge-on interface. Accordingly, the iDPC image is obtained from the selected area designated by the red square in Figure 2C (from the same region where the distinct ellipticity pattern is observed) and presented in Figure 3A. Thorough postprocessing image analysis is applied to this image to unravel its reference structure. In Figure 3A, one can observe that in (∼2 nm) thin layers, some atomic columns are clearly elliptic, while the other alternating ones in a direction parallel to the interface are more localized and rounder. We now refer to specific structures already shown in Figure 1, where the M2 (A) polymorph exhibits exactly this ellipticity pattern for only every second V atomic column parallel to the interface in [100]_R_ projection. Accordingly, the simulated iDPC image associated with the M2 (A) phase matches well the experimental image in panel B of Figure 3. The quantifications of the structures from both the experiment and simulation (Figure 3C) converge well and indicate the symmetrical bond distribution. In addition, the two distinct peaks appearing in the histogram (Figure 3D) further confirm the symmetrical bonds in accordance with the theoretical values of the projected M2 (A) structure. As Figure 3E,F specifies, the blue atomic column (i.e., V1) uniformly shows a half-peak intensity value of 107 pm in an out-of-plane direction across the atomic column diameter, whereas the red atomic column (V2) exhibits a value of 146 pm in this direction. This evidently implies that clear ellipticity occurs for the V2 atomic columns, whereas it is absent for the V1 atomic columns. It is interesting to note that, as it was found in Figure 1C, the lateral oxygen atoms in [100]_R_ projection of M2 [i.e., M2 (A)] also display a specific pattern of directional ellipticity. This subtle point is confirmed by experimental observation given in Figure 3F, where for the O1 atomic column, the intensity length (78 pm) is considerably larger in the out-of-plane direction than that of the O2 (60 pm). These quantifications with their specific directionalities strongly confirm the coexistence of the intermediate M2 phase in a few nanometer-thick layers of the VO_2_ film. In other words, the transitional domain (i.e., zone III) right above the intermixing region is dominated by the intermediate M2 (A) phase (see also Figure S6). In particular, a polymorph that exhibits ellipticity every second V atomic column (and also ellipticity in specific O atomic columns) and possesses symmetrical bonds (two V–O distance peaks) with no V–V dimerization is indeed the M2 monoclinic phase discovered here. Note that we have not identified the other expected orientation of M2 [i.e., M2 (B)] by the STEM imaging, yet for completeness, we have included this orientation in our framework (Figure 1) to clarify that also this projection direction must be present.

Figure 3G–L illustrates the images associated with a region of the VO_2_ film where both V atomic columns show tilted ellipticity (see Figure 3K) and oxygen atoms are completely circular (see Figure 3L). The agreement between simulated and experimental quantification results demonstrates the presence of relatively symmetrical V–O bonds. These observations fit well with another orientation of M1, namely, M1 (B) in [010]_R_ projection. In contrast to previous phases, the strong V atom ellipticity in M1 (B) results in a wide spread of the collected data points. In other words, the larger the atomic ellipticity, the less precisely defined the atomic center of mass becomes during image analysis, ultimately leading to more scattered O atom positions. Nevertheless, as Figure 3J demonstrates, despite this scattering, the two distinct peaks confirm the symmetrical bonds of the M1 (B) phase orientation.

In addition to the local atomic-scale investigations offered above, X-ray diffraction (XRD) reciprocal space mapping hints at the formation of the intermediate phases (in particular M2) within the VO_2_ thin films on the TiO_2_ (001) substrate in a more global picture: see Figure S7. The intermixing region (zone II) in Figure 1C and the long tails revealed in the reciprocal space map (RSM) in Figure S7A suggest the formation of some other intermediate phase(s) (e.g., T polymorph) as well.^29^ However, this topic should be scrutinized in a separate focused study. The double stretched tails observed in the RSM of VO_2_ [002] reflection (seen Figure S7A) demonstrate a pronounced strain gradient in the film as was also suggested by Rodríguez et al.^29^ The strain state and its relationship with the transitory domain of the film are scrutinized next.

**Figure 4:**
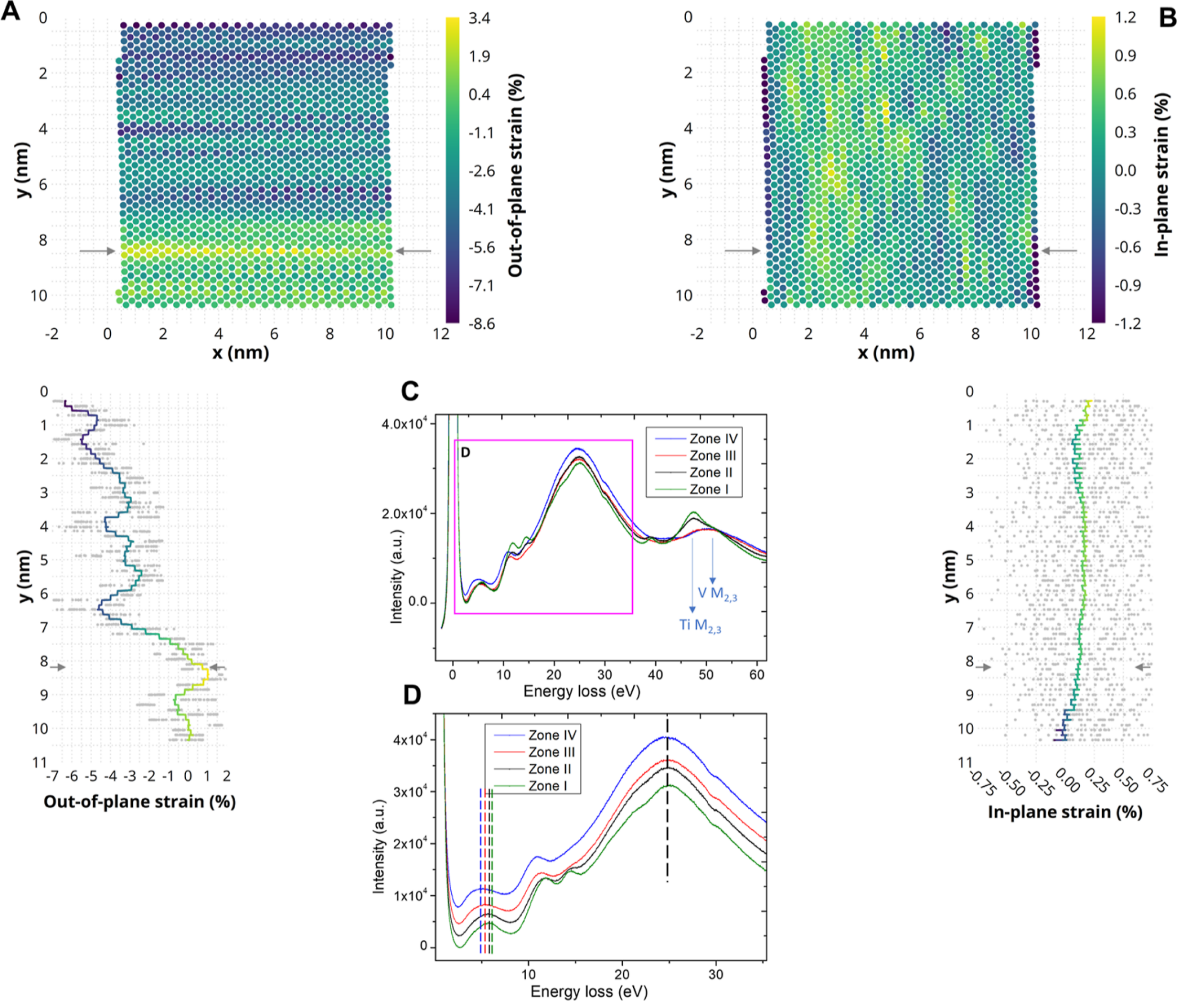
Atomic structure strain mapping and electron energy loss spectroscopy (EELS) analysis. (A) Strain analysis along the growth direction [001]_R_ (out-of-plane) showing the compressive strain values in the VO_2_ film (the arrows indicate the interface between the TiO_2_ substrate and VO_2_ film), (B) strain analysis along the in-plane direction [110]_R_ of the film, revealing a tensile strain generated in the VO_2_ film, (C) EELS spectra recorded from the cross-section lamella of VO_2_ on TiO_2_ (001) with projection [100] near the zero-loss peak, and (D) higher resolution of the spectra from the zero-loss peak to the plasmon region, with equal vertical offsets for clarity.

Up to this point, the coexistence of the various VO_2_ polymorphs, in particular, the M2 phase, has been resolved directly by atomic structure electron microscopy observations. Nevertheless, it is required to get some insights into the potential physical origins of phase coexistence during MIT, specifically the stabilization of the intermediate M2 phase. It has been suggested in the literature that the intermediate phases can be stabilized by strain, doping, and electronic excitation.^36−38^ Nevertheless, due to the lack of atomically resolved information, it has been a persistent challenge to correlate strain locally to the domains of the structural polymorphs. We aim to address this by mapping the strain in cross sections along the growth direction (*c*-axis, out-of-plane) and the in-plane direction of the film. To this purpose, atomic-scale strain measurements using real-space STEM images are accomplished. Figure 4A,B displays the strain distributions normal and parallel to the (edge-on) interface. These results cover zones I, II, and III right below the fully dimerized M1 phase region depicted in Figure 2C. The corresponding iDPC image for the strain analysis is provided in Figure S8. As can be perceived from Figure 4A,B, the absolute strain values in the film are compressive and tensile in the growth direction and in-plane direction, respectively. In particular, in the growth direction (*y*-axis), one can notice a strong strain gradient, compressive in nature. Indeed, zone I (TiO_2_ (001) substrate) as the reference exhibits an almost zero strain value. As the strain profile in Figure 4 denotes, by moving from the substrate to the film in the out-of-plane direction, the strain becomes increasingly more compressive (negative values). An interesting sharp tensile strain (indicated by the arrows) can be observed exactly at the TiO_2_–VO_2_ interface induced by the lattice mismatch. Nevertheless, some local fluctuations of strain in the atomic layers are also noticeable in the out-of-plane direction in the VO_2_ film. The fluctuating strain values predominantly correlate with the M2 polymorph domain (in zone III). This seems to be in line with the observed strain behavior reported by Rodríguez et al.^29^ that can be attributed to the formation of the intermediate phases in VO_2_ films. Due to Poisson’s ratio, obviously, a tensile strain must be present along the in-plane direction [110] of the film as Figure 4 suggests. Thus, the stabilization of the M2 phase at room temperature can probably be attributed to the prevalent strain gradient generated within the VO_2_ thin film on the TiO_2_ substrate upon cooling from a high temperature.

In addition to the studied structural aspects, Figure 4C,D demonstrates EELS analysis across the film to understand the local electronic properties and the band gap characteristics.

Electron energy loss spectra were recorded in the low-loss region, from the zero-loss peak to beyond the Ti and V M_2,3_ absorption edges as shown in Figure 4C,D. The spectra were recorded in the four different zones I to IV as indicated in Figure 2c in different parts of the sample. The Ti and V M_2,3_ edges are important to confirm that zone II is indeed an intermixed region, because the intensity of the Ti M_2,3_ is appreciable, but that zone III is effectively the same as zone IV which must be rather pure VO_2_. The peaks in the range of 0–30 eV before the M_2,3_ edges arise from the collective excitations from the valence bands to the conduction bands of the respective sample layers. When (the maxima in) the zero-loss peaks are aligned well, the maximum intensities in the main plasmon peak at about 25 eV are also aligned well for all four zones, as indicated by the vertical dashed black line in Figure 4D. However, systematic differences are observed in the position of the first peak after the zero-loss peak at about 5 eV that can be associated with states close to the band gap (E_g_). Indeed, in all different parts of the films where we recorded spectra, always a systematic trend was observed that this peak moved to increasingly lower energies when going from zone I via II and III to zone IV. This suggests that the effective E_g_ reduces systematically when going from zone I to IV. However, a more accurate method to determine the value of the band gap is to extrapolate the linear fit to the slope of the low energy side of this peak toward lower energy and then the intercept of this linear fit with the horizontal axis is a measure of the band gap. The exact procedure we performed to do this is described in detail in Supporting Information.

Quantifying the band gap using this procedure, it was found to be 2.89 eV for the pure TiO_2_ substrate (zone I), 2.65 eV in zone II (intermixed layer), 2.43 eV in zone III (M2 containing layer), and finally 1.92 eV in the M1 region (zone IV). The value we measured for rutile TiO_2_ 2.9 eV is rather close to its known value of 3.0–3.1 eV.^39,40^ The value for the M1 phase of VO_2_ 1.9 eV is somewhat larger than reported in some earlier works.^41,42^ Note that in the different parts of the sample analyzed, the E_g_ values we derived differ by about 0.2–0.3 eV but that the trend was always very systematic, i.e., E_g_ of zone I > zone II > zone III > zone IV. This shows that a significant change in the band gap occurs for the layers and also allows us to differentiate between the zones III and IV and thus between the M1 and M2 monoclinic layers of the sample. Note that zone IV can be considered a pure M1 phase and that in zone III, the M2 phase is present but not exclusively since in addition some M1 occurs also due to the overlap that occurs in the projection direction in the relative thick TEM sample as produced by the focused ion beam. The actual difference in band gap between the M2 and M1 phase in our sample is therefore larger than the values we measured here for the difference in E_g_ between zones III and IV.

## Conclusions

In summary, we have studied the cross section of epitaxially grown VO_2_ films on TiO_2_ (001) using atomic-resolution STEM. Using iDPC, we have directly resolved both V and O atomic columns simultaneously and observed the presence of the intermediate monoclinic M2 phase, whose occurrence is strain-related, in thin nanolayers (∼2 nm) above the interface. We have distinguished this M2 phase from the main monoclinic M1 phase using a set of criteria involving the symmetry of bonds and the ellipticity of atoms (both cations and anions). The methodology developed here based on state-of-the-art STEM images in which also the oxygen atomic columns are resolved accurately (like possible with iDPC images) and in which also the ellipticity of projected atomic columns can be quantified, can be applied to a wide range of oxides or materials containing a mixture of low and high *Z* atomic columns. This is particularly important for improving our understanding of the atomic structures of materials varying at nanometer length scales that cannot be captured with methods that analyze materials on a more global scale like diffraction-based techniques.

The comprehensive detection of the different zones in the VO_2_ film (especially the M2 domain) can potentially have a significant effect on the design of modern optoelectronics and memristors. Being able to resolve the spatial configuration of the M2 (and M1) phases provides the prospect to tune the amount and distribution of such intermediate phases in order to optimize the electronic properties within the VO_2_ films.

## Experimental Methods

### Pulsed Laser Deposition

Epitaxial VO_2_ films were grown on single-crystal rutile TiO_2_ (001) substrates (lattice constants *a* = *b* = 0.459 nm, *c* = 0.296 nm) from CrysTec GmbH (Germany). The substrates were cleaned by ultrasonication in ethanol, IPA, and DI water for 10 min each before being dried with a nitrogen gun to clean the surface thoroughly. The PLD method was used to deposit the film, in which a KrF excimer laser (λ = 248 nm) was focused on a vanadium metal target (99.9% pure) with a fluence of ∼2.5 J cm^–2^ and pulse rate of 10 Hz. Oxygen pressure (P_O_2__) inside the chamber was kept constant at 7.5 mTorr (∼0.0099 mbar) and substrate temperature was varied from 500 to 350 °C, resulting in stoichiometric VO_2_ films of varying film and intermediate layer thicknesses.

### Specimen Preparation for Electron Microscopy

TEM lamellas were made from the samples using an FEI Helios G4 CX DualBeam system with a Ga-focused ion beam and thinned to electron transparency using the ion beam with progressively lower accelerating voltages and current doses. The specimen was then cleaned using argon plasma for 5 min before inserting into the microscope.

### Scanning Transmission Electron Microscopy

A probe- and image-corrected Thermo Fisher Scientific Themis Z scanning transmission electron microscope equipped with a Dual-X energy-dispersive X-ray spectrometer was operated at 300 kV for atomic structure imaging. Aberrations were corrected up to the fourth order resulting in a point resolution of ∼65 pm, while the convergence semiangle was set as 24 mrad. The STEM images obtained were filtered in Velox software to reduce the background noise and subsequently analyzed.

### XRD and Reciprocal Space Mapping

The crystal structure of the film was analyzed using high-resolution XRD equipment (Panalytical X’pert Pro MRD), which was operated to perform 2θ–ω scans and reciprocal space mapping of the thin films.

### Electron Energy Loss Spectroscopy

Cross-section lamellas from the samples were prepared and taken to the Ernst Ruska-Centre for Microscopy and Spectroscopy with Electrons (ER-C) and examined using a Hitachi HF5000 probe-corrected scanning transmission electron microscope with a cold field emission gun operated at 200 kV with a CEOS CEFID energy filter, giving an energy resolution of 500 meV at fwhm. Electron energy loss spectra were taken near the low-loss region from the zero-loss peak up to beyond the M_2,3_ edges and near the V and O core-loss region. We used dispersions of 8, 16, and 64 eV on a 4096 × 4096 pixelated TVIPS XF416 detector. The eV/pixel can be calculated by dividing the dispersion with the number of pixels of the detector. The corresponding energies per pixel then correspond to 0.002, 0.004, and 0.015 eV/px.

## Supporting Information Available

Schematic of the PLD setup, scanning electron microscope image of the VO_2_ film, method of calculation of theoretical bond distances for nonelliptical/circular atoms and elliptical atoms, with calculated values, steps of lamella preparation for STEM, structure and lattice parameters of the structures for simulations, with simulated images, steps for bond length analysis, XRD RSMs of the film, ellipticity and strain mapping analysis image, atomic structure of the cross section of the film, and EELS analysis (PDF)

## References

[ref1] Sood A.; Shen X.; Shi Y.; Kumar S.; Park S. J.; Zajac M.; Sun Y.; Chen L.-Q.; Ramanathan S.; Wang X.; Chueh W. C.; Lindenberg A. M. Universal Phase Dynamics in VO_2_ Switches Revealed by Ultrafast Operando Diffraction. Science 2021, 373 (6552), 352–355. 10.1126/science.abc0652. 34437156

[ref2] Li G.; Xie D.; Zhong H.; Zhang Z.; Fu X.; Zhou Q.; Li Q.; Ni H.; Wang J.; Guo E.; He M.; Wang C.; Yang G.; Jin K.; Ge C. Photo-Induced Non-Volatile VO_2_ Phase Transition for Neuromorphic Ultraviolet Sensors. Nat. Commun. 2022, 13 (1), 1729 10.1038/s41467-022-29456-5. 35365642 PMC8975822

[ref3] Wall S.; Yang S.; Vidas L.; Chollet M.; Glownia J. M.; Kozina M.; Katayama T.; Henighan T.; Jiang M.; Miller T. A.; Reis D. A.; Boatner L. A.; Delaire O.; Trigo M. Ultrafast Disordering of Vanadium Dimers in Photoexcited VO_2_. Science 2018, 362 (6414), 572–576. 10.1126/science.aau3873. 30385575

[ref4] Yuan R.; Duan Q.; Tiw P. J.; Li G.; Xiao Z.; Jing Z.; Yang K.; Liu C.; Ge C.; Huang R.; Yang Y. A Calibratable Sensory Neuron Based on Epitaxial VO_2_ for Spike-Based Neuromorphic Multisensory System. Nat. Commun. 2022, 13 (1), 3973 10.1038/s41467-022-31747-w. 35803938 PMC9270461

[ref5] Pouget J.-P. Basic Aspects of the Metal-Insulator Transition in Vanadium Dioxide VO_2_: A Critical Review. C. R. Phys. 2021, 22 (1), 37–87. 10.5802/crphys.74.

[ref6] Johnson A. S.; Perez-Salinas D.; Siddiqui K. M.; Kim S.; Choi S.; Volckaert K.; Majchrzak P. E.; Ulstrup S.; Agarwal N.; Hallman K.; Haglund R. F.; Günther C. M.; Pfau B.; Eisebitt S.; Backes D.; Maccherozzi F.; Fitzpatrick A.; Dhesi S. S.; Gargiani P.; Valvidares M.; Artrith N.; de Groot F.; Choi H.; Jang D.; Katoch A.; Kwon S.; Park S. H.; Kim H.; Wall S. E. Ultrafast X-Ray Imaging of the Light-Induced Phase Transition in VO_2_. Nat. Phys. 2022, 19 (2), 215–220. 10.1038/s41567-022-01848-w.

[ref7] Wentzcovitch R. M.; Schulz W. W.; Allen P. B. VO_2_: Peierls or Mott-Hubbard? A View from Band Theory. Phys. Rev. Lett. 1994, 72 (21), 3389–3392. 10.1103/PhysRevLett.72.3389. 10056186

[ref8] Biermann S.; Poteryaev A.; Lichtenstein A. I.; Georges A. Dynamical Singlets and Correlation-Assisted Peierls Transition in VO_2_. Phys. Rev. Lett. 2005, 94 (2), 26404 10.1103/PhysRevLett.94.026404. 15698203

[ref9] Cavalleri A.; Dekorsy Th.; Chong H. H. W.; Kieffer J. C.; Schoenlein R. W. Evidence for a Structurally-Driven Insulator-to-Metal Transition in VO_2_: A View from the Ultrafast Timescale. Phys. Rev. B: Condens. Matter Mater. Phys. 2004, 70 (16), 161102 10.1103/PhysRevB.70.161102.

[ref10] Guzmán-Verri G. G.; Brierley R. T.; Littlewood P. B. Cooperative Elastic Fluctuations Provide Tuning of the Metal-Insulator Transition. Nature 2019, 576 (7787), 429–432. 10.1038/s41586-019-1824-9. 31853079

[ref11] Kumar S.; Strachan J. P.; Pickett M. D.; Bratkovsky A.; Nishi Y.; Williams R. S. Sequential Electronic and Structural Transitions in VO_2_ Observed Using X-Ray Absorption Spectromicroscopy. Adv. Mater. 2014, 26 (44), 7505–7509. 10.1002/adma.201402404. 25319233

[ref12] Park J. H.; Coy J. M.; Kasirga T. S.; Huang C.; Fei Z.; Hunter S.; Cobden D. H. Measurement of a Solid-State Triple Point at the Metal-Insulator Transition in VO_2_. Nature 2013, 500 (7463), 431–434. 10.1038/nature12425. 23969461

[ref13] Lee D.; Chung B.; Shi Y.; Kim G.-Y.; Campbell N.; Xue F.; Song K.; Choi S.-Y.; Podkaminer J. P.; Kim T. H.; Ryan P. J.; Kim J.-W.; Paudel T. R.; Kang J.-H.; Spinuzzi J. W.; Tenne D. A.; Tsymbal E. Y.; Rzchowski M. S.; Chen L. Q.; Lee J.; Eom C. B. Isostructural Metal-Insulator Transition in VO_2_. Science 2018, 362 (6418), 1037–1040. 10.1126/science.aam9189. 30498123

[ref14] Chen F. H.; Fan L. L.; Chen S.; Liao G. M.; Chen Y. L.; Wu P.; Song L.; Zou C. W.; Wu Z. Y. Control of the Metal-Insulator Transition in VO_2_ Epitaxial Film by Modifying Carrier Density. ACS Appl. Mater. Interfaces 2015, 7 (12), 6875–6881. 10.1021/acsami.5b00540. 25751594

[ref15] Yang M.; Yang Y.; Hong B.; Wang L.; Hu K.; Dong Y.; Xu H.; Huang H.; Zhao J.; Chen H.; Song L.; Ju H.; Zhu J.; Bao J.; Li X.; Gu Y.; Yang T.; Gao X.; Luo Z.; Gao C. Suppression of Structural Phase Transition in VO_2_ by Epitaxial Strain in Vicinity of Metal-Insulator Transition. Sci. Rep. 2016, 6 (1), 23119 10.1038/srep23119. 26975328 PMC4792152

[ref16] Jeong J.; Aetukuri N.; Graf T.; Schladt T. D.; Samant M. G.; Parkin S. S. P. Suppression of Metal-Insulator Transition in VO_2_ by Electric Field-Induced Oxygen Vacancy Formation. Science 2013, 339 (6126), 1402–1405. 10.1126/science.1230512. 23520104

[ref17] Lu Q.; Bishop S. R.; Lee D.; Lee S.; Bluhm H.; Tuller H. L.; Lee H. N.; Yildiz B. Electrochemically Triggered Metal-Insulator Transition between VO_2_ and V_2_O_5_. Adv. Funct. Mater. 2018, 28 (34), 1803024 10.1002/adfm.201803024.

[ref18] Lee Y. J.; Hong K.; Na K.; Yang J.; Lee T. H.; Kim B.; Bark C. W.; Kim J. Y.; Park S. H.; Lee S.; Jang H. W. Nonvolatile Control of Metal-Insulator Transition in VO_2_ by Ferroelectric Gating. Adv. Mater. 2022, 34 (32), 2203097 10.1002/adma.202203097. 35713476

[ref19] Gurunatha K. L.; Sathasivam S.; Li J.; Portnoi M.; Parkin I. P.; Papakonstantinou I. Combined Effect of Temperature Induced Strain and Oxygen Vacancy on Metal-Insulator Transition of VO_2_ Colloidal Particles. Adv. Funct. Mater. 2020, 30 (49), 2005311 10.1002/adfm.202005311.

[ref20] Han K.; Wu L.; Cao Y.; Wang H.; Ye C.; Huang K.; Motapothula M.; Xing H.; Li X.; Qi D.-C.; Li X.; Renshaw Wang X. Enhanced Metal-Insulator Transition in Freestanding VO_2_ Down to 5 nm Thickness. ACS Appl. Mater. Interfaces 2021, 13 (14), 16688–16693. 10.1021/acsami.1c01581. 33793182

[ref21] Yajima T.; Nishimura T.; Tanaka T.; Uchida K.; Toriumi A. Modulation of VO_2_ Metal-Insulator Transition by Ferroelectric HfO_2_ Gate Insulator. Adv. Electron. Mater. 2020, 6 (5), 1901356 10.1002/aelm.201901356.

[ref22] Strelcov E.; Lilach Y.; Kolmakov A. Gas Sensor Based on Metal-Insulator Transition in VO_2_ Nanowire Thermistor. Nano Lett. 2009, 9 (6), 2322–2326. 10.1021/nl900676n. 19507888

[ref23] Asayesh-Ardakani H.; Nie A.; Marley P. M.; Zhu Y.; Phillips P. J.; Singh S.; Mashayek F.; Sambandamurthy G.; Low K.; Klie R. F.; Banerjee S.; Odegard G. M.; Shahbazian-Yassar R. Atomic Origins of Monoclinic-Tetragonal (Rutile) Phase Transition in Doped VO_2_ Nanowires. Nano Lett. 2015, 15 (11), 7179–7188. 10.1021/acs.nanolett.5b03219. 26457771

[ref24] Sharma Y.; Holt M. V.; Laanait N.; Gao X.; Ivanov I. N.; Collins L.; Sohn C.; Liao Z.; Skoropata E.; Kalinin S. V.; Balke N.; Eres G.; Ward T. Z.; Lee H. N. Competing Phases in Epitaxial Vanadium Dioxide at Nanoscale. APL Mater. 2019, 7 (8), 081127 10.1063/1.5115784.

[ref25] Laverock J.; Jovic V.; Zakharov A. A.; Niu Y. R.; Kittiwatanakul S.; Westhenry B.; Lu J. W.; Wolf S. A.; Smith K. E. Observation of Weakened V–V Dimers in the Monoclinic Metallic Phase of Strained VO_2_. Phys. Rev. Lett. 2018, 121 (25), 256403 10.1103/PhysRevLett.121.256403. 30608778

[ref26] Grandi F.; Amaricci A.; Fabrizio M. Unraveling the Mott-Peierls Intrigue in Vanadium Dioxide. Phys. Rev. Res. 2020, 2 (1), 013298 10.1103/PhysRevResearch.2.013298.

[ref27] Sandiumenge F.; Rodríguez L.; Pruneda M.; Magén C.; Santiso J.; Catalan G. Metallic Diluted Dimerization in VO_2_ Tweeds. Adv. Mater. 2021, 33 (9), 2004374 10.1002/adma.202004374. 33501746

[ref28] Marezio M.; McWhan D. B.; Remeika J. P.; Dernier P. D. Structural Aspects of the Metal-Insulator Transitions in Cr-Doped VO_2_. Phys. Rev. B: Solid State 1972, 5 (7), 2541–2551. 10.1103/PhysRevB.5.2541.

[ref29] Rodríguez L.; Sandiumenge F.; Frontera C.; Caicedo J. M.; Padilla J.; Catalán G.; Santiso J. Strong Strain Gradients and Phase Coexistence at the Metal-Insulator Transition in VO_2_ Epitaxial Films. Acta Mater. 2021, 220, 117336 10.1016/j.actamat.2021.117336.

[ref30] Yang Z.; Ko C.; Ramanathan S. Oxide Electronics Utilizing Ultrafast Metal-Insulator Transitions. Annu. Rev. Mater. Res. 2011, 41 (1), 337–367. 10.1146/annurev-matsci-062910-100347.

[ref31] Zhang Y.-Q.; Chen K.; Shen H.; Wang Y.-C.; Hedhili M. N.; Zhang X.; Li J.; Shan Z.-W. Achieving Room-Temperature M2-Phase VO_2_ Nanowires for Superior Thermal Actuation. Nano Res. 2021, 14 (11), 4146–4153. 10.1007/s12274-021-3355-6.

[ref32] Brito W. H.; Aguiar M. C. O.; Haule K.; Kotliar G. Metal-Insulator Transition in VO_2_: ADFT+DMFTPerspective. Phys. Rev. Lett. 2016, 117 (5), 56402 10.1103/PhysRevLett.117.056402. 27517782

[ref33] Liu M.; Xie S.; Wei L.; Galluzzi M.; Li Y.; Wang Q.; Zhou X.; Wang Y.; Li J. Quantitative Functional Imaging of VO_2_ Metal-Insulator Transition through Intermediate M2 Phase. Acta Mater. 2020, 195, 720–727. 10.1016/j.actamat.2020.06.014.

[ref34] Kim H.; Slusar T. V.; Wulferding D.; Yang I.; Cho J.-C.; Lee M.; Choi H. C.; Jeong Y. H.; Kim H.-T.; Kim J. Direct Observation of the M2 Phase with Its Mott Transition in a VO_2_ Film. Appl. Phys. Lett. 2016, 109 (23), 233104 10.1063/1.4971848.

[ref35] Atul A.; Ahmadi M.; Koutsogiannis P.; Zhang H.; Kooi B. J. Strong Substrate Influence on Atomic Structure and Properties of Epitaxial VO_2_ Thin Films. Adv. Mater. Interfaces 2024, 11, 2300639 10.1002/admi.202300639.

[ref36] Strelcov E.; Tselev A.; Ivanov I.; Budai J. D.; Zhang J.; Tischler J. Z.; Kravchenko I.; Kalinin S. V.; Kolmakov A. Doping-Based Stabilization of the M2 Phase in Free-Standing VO_2_ Nanostructures at Room Temperature. Nano Lett. 2012, 12 (12), 6198–6205. 10.1021/nl303065h. 23145774

[ref37] Liu K.; Lee S.; Yang S.; Delaire O.; Wu J. Recent Progresses on Physics and Applications of Vanadium Dioxide. Mater. Today 2018, 21 (8), 875–896. 10.1016/j.mattod.2018.03.029.

[ref38] Shao Z.; Cao X.; Luo H.; Jin P. Recent Progress in the Phase-Transition Mechanism and Modulation of Vanadium Dioxide Materials. NPG Asia Mater. 2018, 10 (7), 581–605. 10.1038/s41427-018-0061-2.

[ref39] Sanjinés R.; Tang H.; Berger H.; Gozzo F.; Margaritondo G.; Lévy F. Electronic Structure of Anatase TiO_2_ Oxide. J. Appl. Phys. 1994, 75 (6), 2945–2951. 10.1063/1.356190.

[ref40] Pascual J.; Camassel J.; Mathieu H. Fine Structure in the Intrinsic Absorption Edge of TiO_2_. Phys. Rev. B: Solid State 1978, 18 (10), 5606–5614. 10.1103/PhysRevB.18.5606.

[ref41] Eyert V. VO_2_: A Novel View from Band Theory. Phys. Rev. Lett. 2011, 107 (1), 16401 10.1103/PhysRevLett.107.016401. 21797557

[ref42] Xu S.; Shen X.; Hallman K. A.; Haglund R. F.; Pantelides S. T. Unified Band-Theoretic Description of Structural, Electronic, and Magnetic Properties of Vanadium Dioxide Phases. Phys. Rev. B 2017, 95 (12), 125105 10.1103/PhysRevB.95.125105.

