# Supplementary material for: Atomically Resolved Phase Coexistence in VO2 Thin Films
Source: ACS Nano. 2024 May 16;18(21):13496–505. doi: 10.1021/acsnano.3c10745 (PMC11140831; doi:10.1021/acsnano.3c10745)
Supplement: Supplementary file 1 — nn3c10745_si_001.pdf [file nn3c10745_si_001.pdf]

Supporting Information

Atomically Resolved Phase Coexistence in VO_2 Thin Films

Masoud Ahmadi[1], Atul Atul[1]*, Sytze de Graaf[1], Ewout van der Veer[1], Ansgar Meise[2], Amir Hossein Tavabi[2], Marc Heggen[2], Rafal E. Dunin-Borkowski[2], Majid Ahmadi[1], Bart J. Kooi[1]

[1]*Zernike Institute for Advanced Materials, University of Groningen, Nijenborgh 4, 9747 AG, Groningen, The Netherlands*

[2]*Ernst Ruska-Centre for Microscopy and Spectroscopy with Electrons (ER-C), Forschungszentrum Jülich, 52425 Jülich, Germany*

**Corresponding author: atul061094@gmail.com*

Pulsed Laser Deposition

Fig. S1 demonstrates the schematic representation of the pulsed laser deposition (PLD) process utilized in this study.

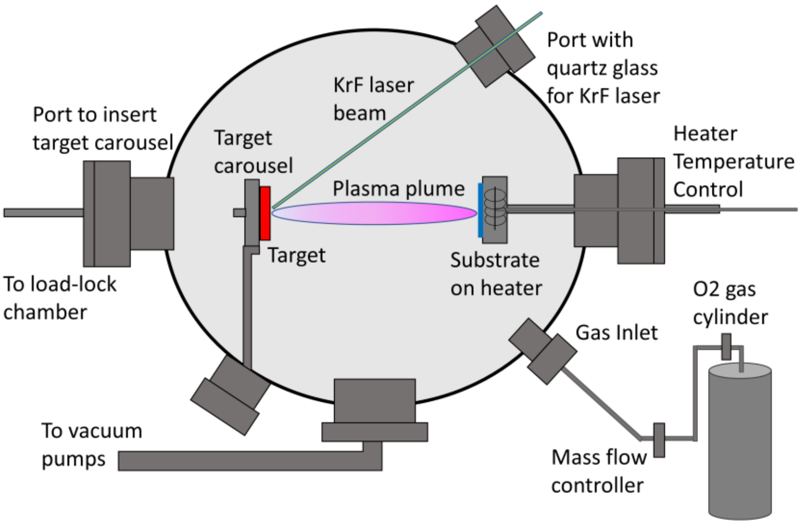

Figure S1. Schematic representation of the PLD process for depositing VO_2 film on TiO_2 substrate.

Scanning electron microscopy

In order to observe the distribution and size of the domains in the deposited VO_2 thin film, a scanning electron microscope (Helios G4 CX Dual-Beam) was used. Fig. S2 shows that the film exhibits mostly rectangular and perpendicular domains along [110] ($a=b\neq c$).

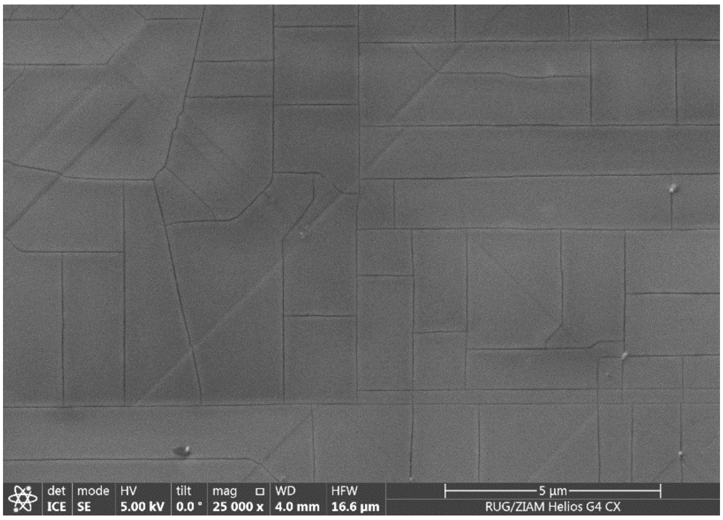

Figure S2. SEM micrograph of the deposited VO_2 film.

Calculation of theoretical bond distances

The theoretical bond distances were obtained from the analysis of the VESTA [1] models of the structures. The structural parameters of R, M1 and M2 phases in this study are attained from the literature [2–5]. The V-O and V-V distances in three dimensions can be achieved from the software itself but we are interested in the actual distances in the projected orientations in order to compare with the STEM images precisely. To find these distances, high-resolution images of the structure were exported and analyzed using Gatan Digital Micrograph software. Two examples of such calculations in different orientations will be described below.

- **Orientation without ellipticity in atomic column**

The atoms are aligned in the column and are observed as circular in STEM images. The center of the titanium atom can be found by making two perpendicular line scans of the width of the atomic column, and the center of the intersecting area gives the atom position. The same process is followed for all the 6 atoms (only a single Ti-O bond is shown in Fig. S3 for clarity) which leads to the 6 Ti-O (V-O) distances and angles.

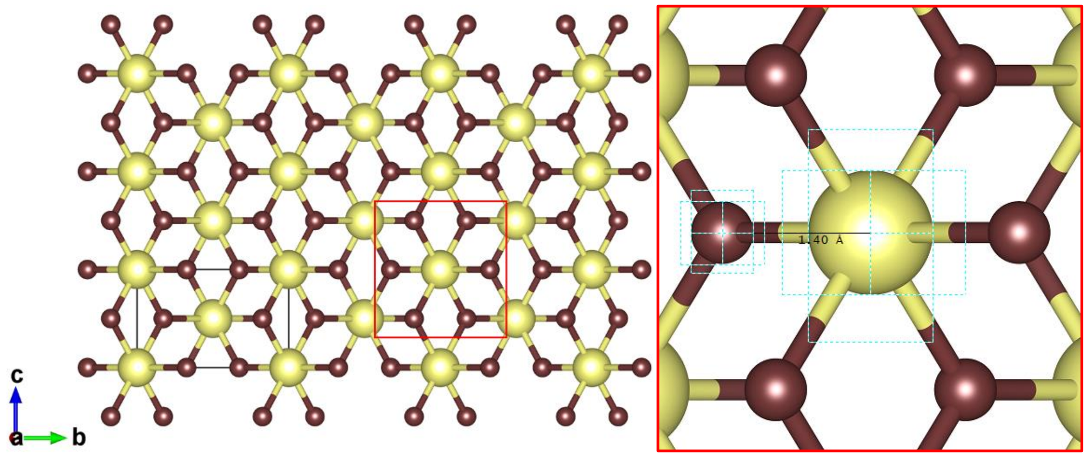

Figure S3. The calculation of theoretical bond distances in TiO_2 R phase.

- **Orientation with ellipticity in atomic column**

In this case, vanadium atoms are not aligned in the column so we see an elliptical shape in STEM images. The average position of the two atoms is taken as the center of the V atom for calculation. This is found again using two perpendicular line scans of the width of the atomic column (of the 2 atoms). The center of the intersecting square area gives the center of the ellipse. The same procedure is performed for the O atoms with ellipticity (only one is shown for clarity), which provides the V-O distances and angles.

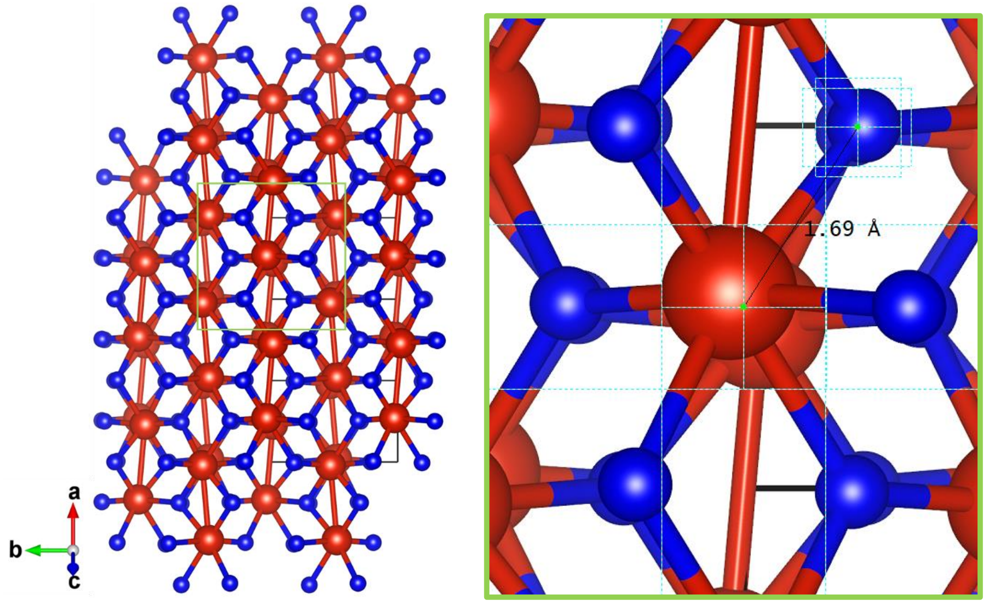

Figure S4. The calculation of theoretical bond distances in VO_2 M1 (B) phase.

Tables S1

Calculated theoretical bond distances

R-phase TiO_2

Orientation: R [100] [001]

		Distance (in Angstrom)	Angle (0 to 360 deg.)
**Ti1**	Ti1-O1	1.39	0
	Ti1-O2	1.72	59.24
	Ti1-O3	1.73	121.64
	Ti1-O4	1.40	180
	Ti1-O5	1.73	238.29
	Ti1-O6	1.72	300.64

M2 (A)-Phase

M2 [100] [010] equivalent to R [100] [001]

		Distance (in Angstrom)	Angles (0 to 360 deg.)
**V1**	V1-O1	1.39	0.04
	V1-O2	1.73	56.52
	V1-O3	1.73	123.61
	V1-O4	1.39	180.32
	V1-O5	1.73	236.27
	V1-O6	1.73	303.54

		Distance (in Angstrom)	Angles (0 to 360 deg.)
**V2**	V2-O1	1.31	-0.20
	V2-O2	1.68	58.78
	V2-O3	1.68	121.42
	V2-O4	1.31	180.20
	V2-O5	1.69	238.83
	V2-O6	1.69	301.02

M1 (B)-Phase

M1 [102] [100] equivalent to R [010] [001]

		Distance (in Angstrom)	Angles (0 to 360 deg.)
**V1**	V1-O1	1.39	-0.80
	V1-O2	1.69	56.26
	V1-O3	1.69	121.30
	V1-O4	1.32	180.40
	V1-O5	1.69	238.16
	V1-O6	1.71	301.88

		Distance (in Angstrom)	Angles (0 to 360 deg.)
**V2**	V2-O1	1.35	1.06
	V2-O2	1.72	58.07
	V2-O3	1.69	122.51
	V2-O4	1.35	180.85
	V2-O5	1.69	238.10
	V2-O6	1.69	302.28

M1 (A)-Phase

M1 [0$\bar{1}$0] [20$\bar{1}$] equivalent to R [$\bar{1}$00] [001]

		Distance (in Angstrom)	Angles (0 to 360 deg.)
**V1**	V1-O1	1.207	-4.25
	V1-O2	1.549	58.76
	V1-O3	1.669	127.50
	V1-O4	1.484	184.55
	V1-O5	1.874	234.84
	V1-O6	1.795	297.58

		Distance (in Angstrom)	Angles (0 to 360 deg.)
**V2**	V2-O1	1.484	4.58
	V2-O2	1.874	54.86
	V2-O3	1.794	117.60
	V2-O4	1.208	175.78
	V2-O5	1.549	238.71
	V2-O6	1.668	307.51

Red: pair 1

Blue: pair 2

Green: pair 3

Purple: pair 4

Orange: pair 5

Black: pair 6

Note: For all the above data, the average values of each bond type is used in the manuscript figures to compare with the experimental bond length values. For the dimerized M1 (A), the twelve measured bond values are grouped in 6 pairs as mentioned above.

Scanning transmission electron microscopy (STEM)

We first used the high-angle annular dark field (HAADF) imaging mode of STEM which detects the electrons scattered at high angles and is based on Z-contrast imaging (the average atomic number of an element scales the brightness of a resolved atom) [6]. The ease of interpretation in HAADF-STEM images provides the possibility to capture the atomic structural changes of the nanostructured materials. Nevertheless, complex correlated oxides exhibit both light and heavy elements simultaneously that cannot be resolved together by HAADF-STEM mode.

Integrated Differential Phase Contrast (iDPC) imaging

The most recent technique that allows imaging the heavy and light elements is the iDPC mode imaging of STEM. This method outperforms the conventional techniques such as annular bright field (ABF) STEM where wave interference may cause undesirable artifacts. In the iDPC-STEM technique, coherently scattered electrons that scatter within the bright-field disk are collected using a four quadrants segmented detector [7]. The predominance and versatility of this technique have been shown on various oxides and hydride systems [8,9]. Therefore, we have employed this technique extensively throughout the study in order to resolve V (Ti) and oxygen atoms effectively.

Specimen preparation for STEM microscopy

Lamella preparation procedure

A clean area on the sample was chosen to make lamella from, with approximately 45° relative to the domains which are along [110] direction. Protective layers of Carbon and Platinum were deposited using e-beam at 5 kV high voltage and at 1.4 nA with thicknesses about 0.2 µm and 0.5 µm, which are necessary to prevent sample damage during the following steps. Ion-beam Platinum was then deposited at 30 kV, 83 pA, with a thickness of about 2 µm to form a thick protective layer. Bulk ion milling was performed at 30 kV, 21 nA, depth = 20 µm at 52° tilt to reveal the form of the lamella. Intermediate milling was performed at 52 ± 1.5° tilt to reduce lamella thickness to around 1-1.5 µm which is required to be able to free the lamella from the backside. A U-shaped cut was made using 9.3 nA to make the lamella free from the sides where the needle manipulator was attached using Pt deposition. The lamella was then cut off from the sample using Si-pass, and the manipulator was carefully retracted. The grid was cleaned using ion beam, and then the lamella was attached to it from the manipulator using multiple Pt-

deposition of 1 µm. After the manipulator was removed, the lamella was then ready for the thinning process.

The thinning process was performed on a Peltier cooled chip to minimize Ga-ion damages. Thinning was carried out using the Ga-ion beam initially at 30 kV high voltage with 0.23 nA at 52 ± 1.5° tilt up to a thickness less than 300 nm. Thinning up to 100 nm was performed at 83 pA at 52 ± 1.2° tilt. Low kV thinning was then done to reduce surface amorphization at 5 kV, 74 pA, depth = 200 nm, 52 ± 5° tilt; and at 2 kV with 43 pA, depth = 30 nm. The thinned lamella was then ready for atomic structure electron microscopy study.

Table S2. The detailed procedure of TEM lamella preparation.

Electron-beam Carbon Deposition	Electron-beam at 5 kV, 1.4 nA, stage tilt = 0°	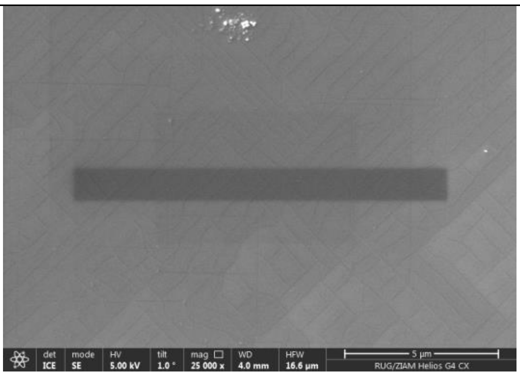
Electron-beam Platinum Deposition	Electron-beam at 5 kV, 1.4 nA, thickness = 0.5 µm, stage tilt = 0°	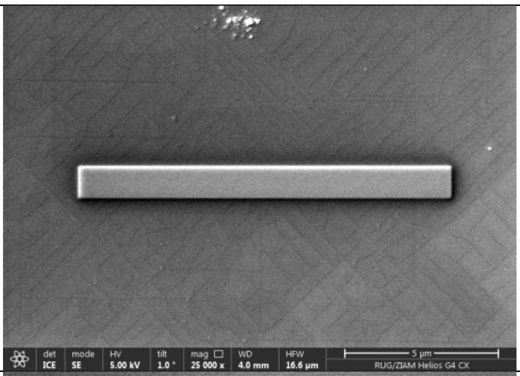
Ion-beam Platinum Deposition	Ion-beam at 30 kV, 83 pA, thickness = 2 µm, stage tilt = 52°	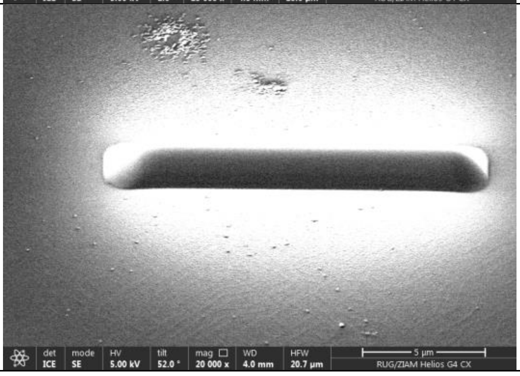

Bulk Ion Milling	Ion-beam at 30 kV, 21 nA, depth = 20 µm, stage tilt = 52°	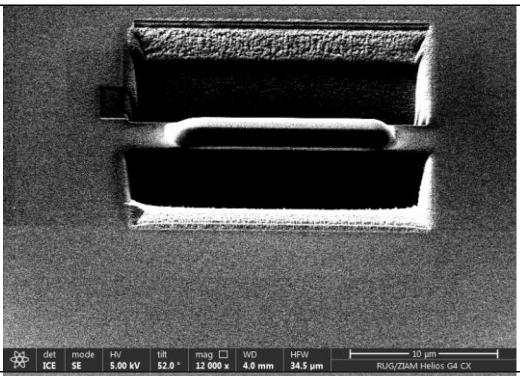
U-shaped cut	Ion-beam at 30 kV, 9.3 nA, stage tilt = 0°	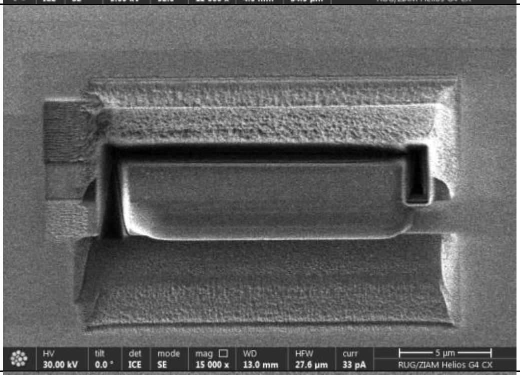
Lift-out	Ion-beam at 30 kV, 2.5 nA, stage tilt = 0°	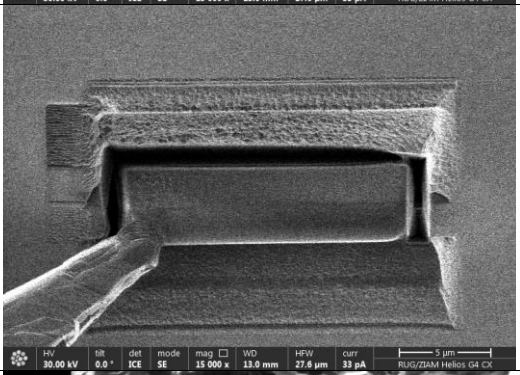
Attachment on the grid with Pt ion-beam	Ion-beam at 30 kV, 2.5 nA, stage tilt = 0°	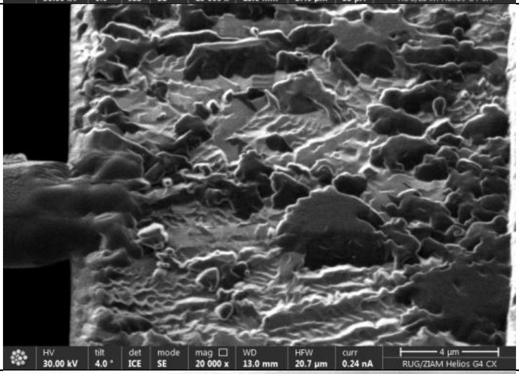
Detaching the needle using Si high-pass	Ion-beam at 30 kV, 2.5 nA, stage tilt = 0°<br><br>This is the lamella attached on the grid, before thinning has been done.	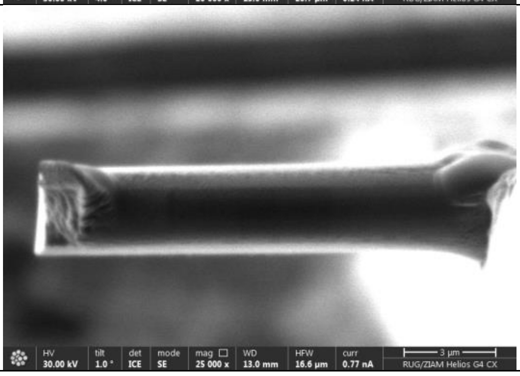

Thinning upto 300 nm	Ion-beam at 30 kV, 2.5 nA, stage tilt = 52 ± 1.5°	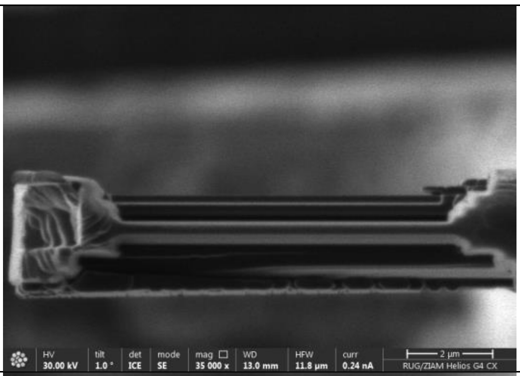
Thinning upto 100 nm	Ion-beam at 30 kV, 83 pA, stage tilt = 52 ± 1.2°	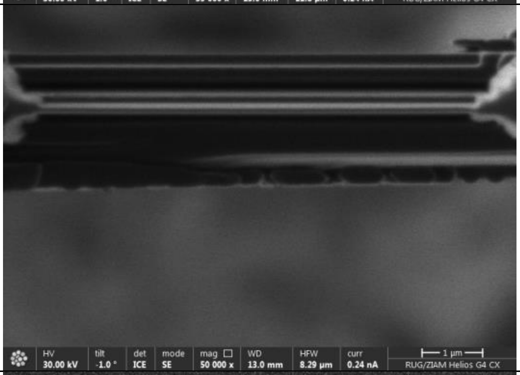
Low kV thinning	Ion-beam at 5 kV, 74 pA, stage tilt = 52 ± 5°	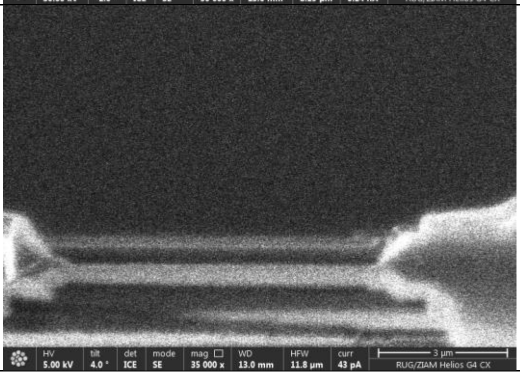

Multi-slice simulations

The VESTA models (.cif files) for the crystal structures of rutile and monoclinic phases were imported into the Dr. Probe software [10] for STEM image simulations. In the performed multi-slice simulations, aberrations were neglected except for the defocus, while the other microscope parameters were set to experimental values accordingly. Debye-Waller factors (B) gained from [2,11,12] were applied in order to account for the atomic thermal vibrations. The thickness of 4-6 nm was found as the optimum condition for simulating the atomic structures of the film. The structural parameters of the simulated phases are given in the following tables. The annular bright field (ABF), annular dark field, BF and HAADF images of the simulated phases are directly obtained from Dr. Probe software and are reported in Fig. S5. Furthermore, for

generating the iDPC images (provided in the main manuscript) from the simulation data, a dedicated MATLAB code was developed and used.

Table S3. TiO_2 structure and lattice parameters for R phase.

#	Atom	x	y	z	B
1	Ti	0	0	0	0.42
2	O	0.3051	0.3051	0	0.60

a (nm)	b (nm)	c (nm)	α (°)	β (°)	γ (°)
0.4593	0.4593	0.2959	90	90	90

Table S4. VO_2 structure and lattice parameters for M1 phase.

#	Atom	x	y	z	B
1	V	0.242	0.975	0.025	0.9
2	O	0.100	0.210	0.200	1.25
3	O	0.390	0.690	0.290	1.25

a (nm)	b (nm)	c (nm)	α (°)	β (°)	γ (°)
0.5753	0.4526	0.5383	90	122.61	90

Table S5. VO_2 structure and lattice parameters for M2 phase.

#	Atom	x	y	z	B
1	V1	0.000	0.265	0.000	1.3
2	V2	0.240	0.000	0.507	1.3
3	O1	0.136	0.235	0.307	1.05
4	O2	0.122	0.000	0.779	1.05
5	O3	0.108	0.500	0.798	1.05

a (nm)	b (nm)	c (nm)	α (°)	β (°)	γ (°)
0.908300	0.576300	0.453200	90	90.3000	90

Table S6. STEM microscope setup in the simulations.

#	Parameter	Value	Units
1	Acceleration Voltage	300	kV
2	Convergence Angle	24	mrad
3	Objective aperture position	0	mrad
4	Defocus	-4	nm

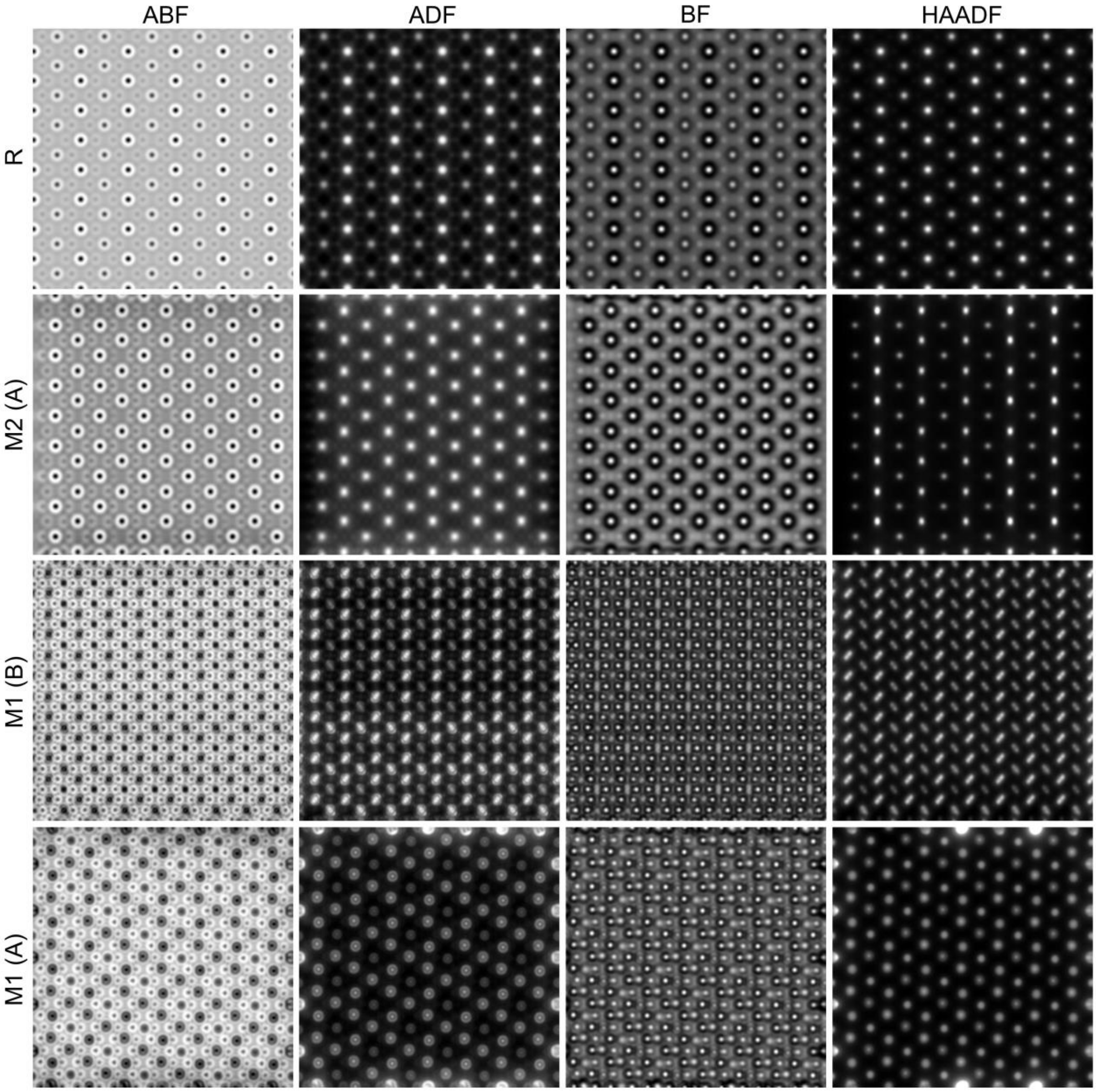

Figure S5. The atomic-structure simulated images of the various phases of VO_2-TiO_2 in different imaging modes. The simulated HAADF-STEM images accurately follow the discussed structural differences (associated with V-atoms) among the polymorphs.

Bond length analysis using an in-house MATLAB script

For proper analysis of the STEM iDPC images, atomic positions need to be calculated accurately. For this purpose, we have developed a post-processing image analysis package using MATLAB. The area required for analysis was cropped and filtered using a Gaussian filter to remove high-frequency noises. Afterward, convolution filtering was carried out which assigns the brightness value of each pixel as a weighted average of the surrounding pixels. This

was performed to make the atom positions as higher intensity as compared to nearby pixels. The script was used to find peaks within a range of brightness values normalized to a maximum of 1. Nearest-neighbor distances (of lattice B atoms with respect to lattice A) as well as angles were calculated by means of an additional script. The image analysis steps for spatial atomic configuration and bond-length determination (here R phase as an example) are given in Table S7. The same procedure was followed for the rest of the iDPC images, i.e. images obtained from experimental microscopy and from simulations.

Table S7. Postprocessing image analysis steps in our developed script.

1	Cropped section from iDPC-STEM image (gaussian-filtered)	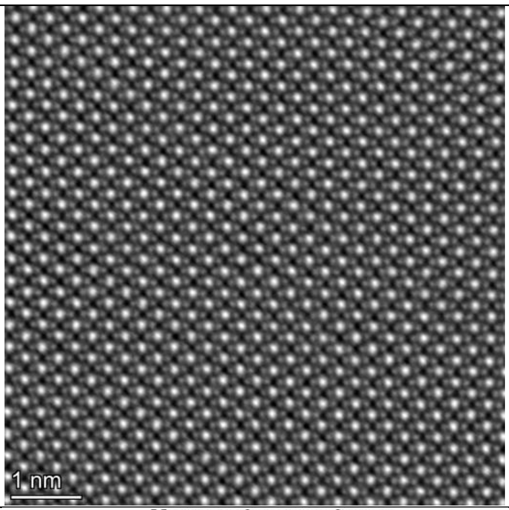
2	Convolution filtering	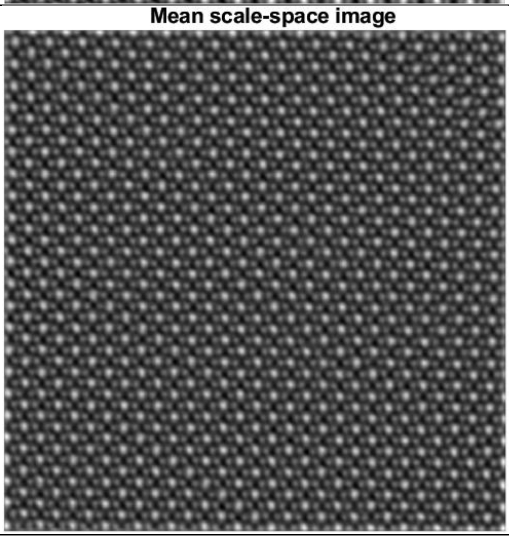

3	Matlab script run to find lattice A atoms (here Ti)		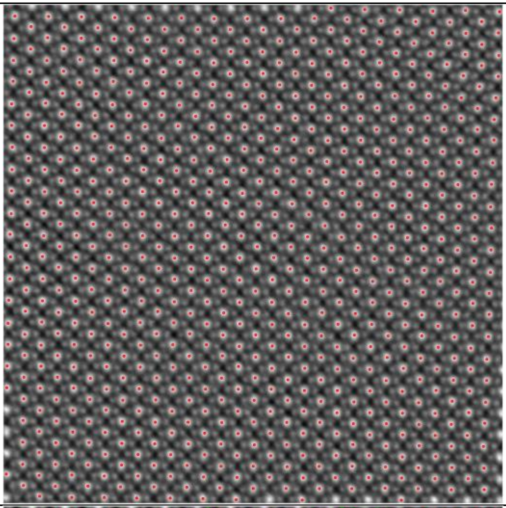
4	Matlab script run to find lattice B atoms (here O)		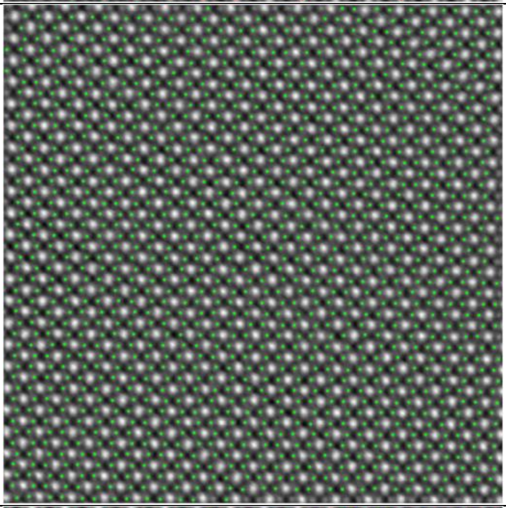
5	Combined A + B lattice image		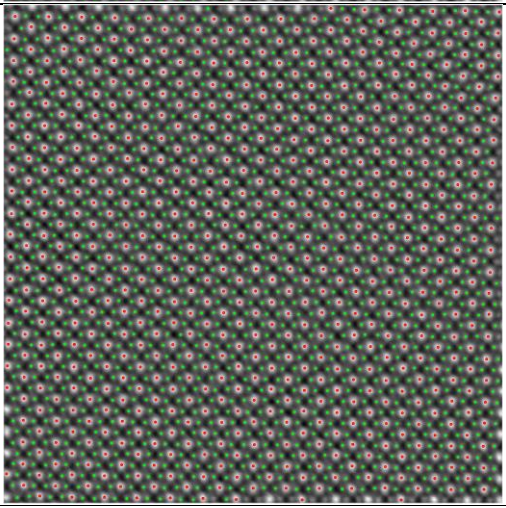

6	Nearest-neighbor distances and angles, spatial configuration	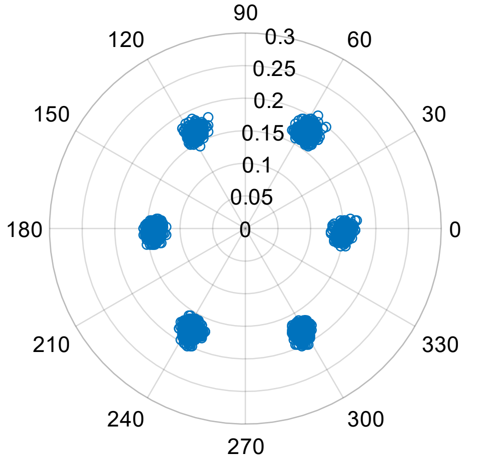
7	Nearest-neighbor distances and intensity histogram	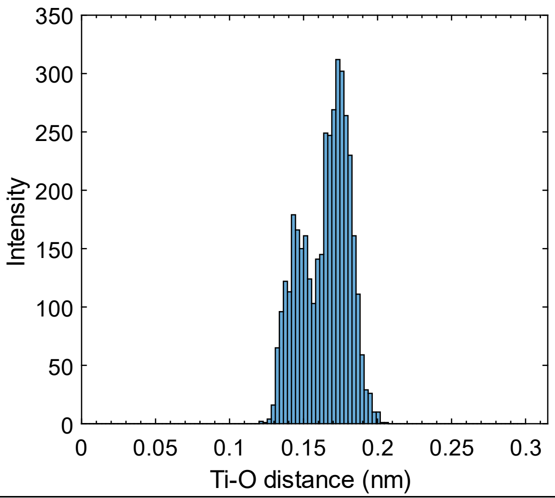

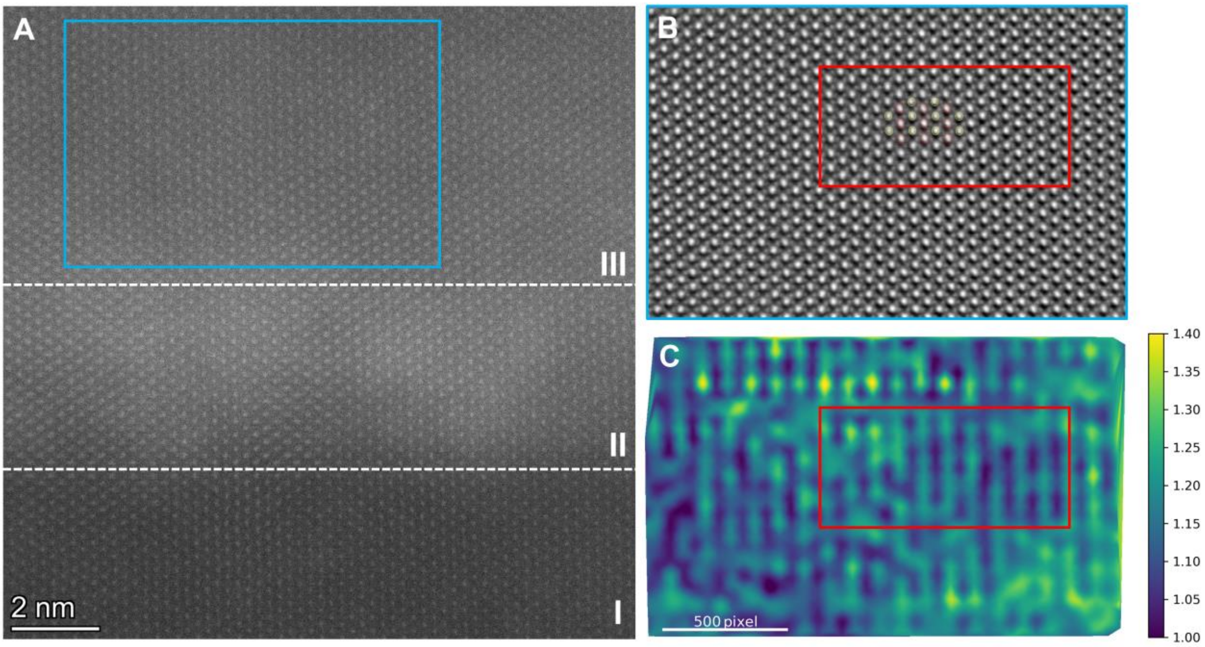

Figure S6. (A) An additional HAADF-STEM image of the VO_2 film on [001] TiO_2 substrate, (B) the iDPC image of a cropped area from Zone III (transitional domain), (C) ellipticity analysis on the cropped area revealing the similar alternating patterns that were already shown in Fig. 2D, approving the dominance of M2 phase in the transitional domain of the film.

X-ray Diffraction and Reciprocal Space Mapping

Fig. S7 depicts the reciprocal space mapping (RSM) results along different orientations of the film.

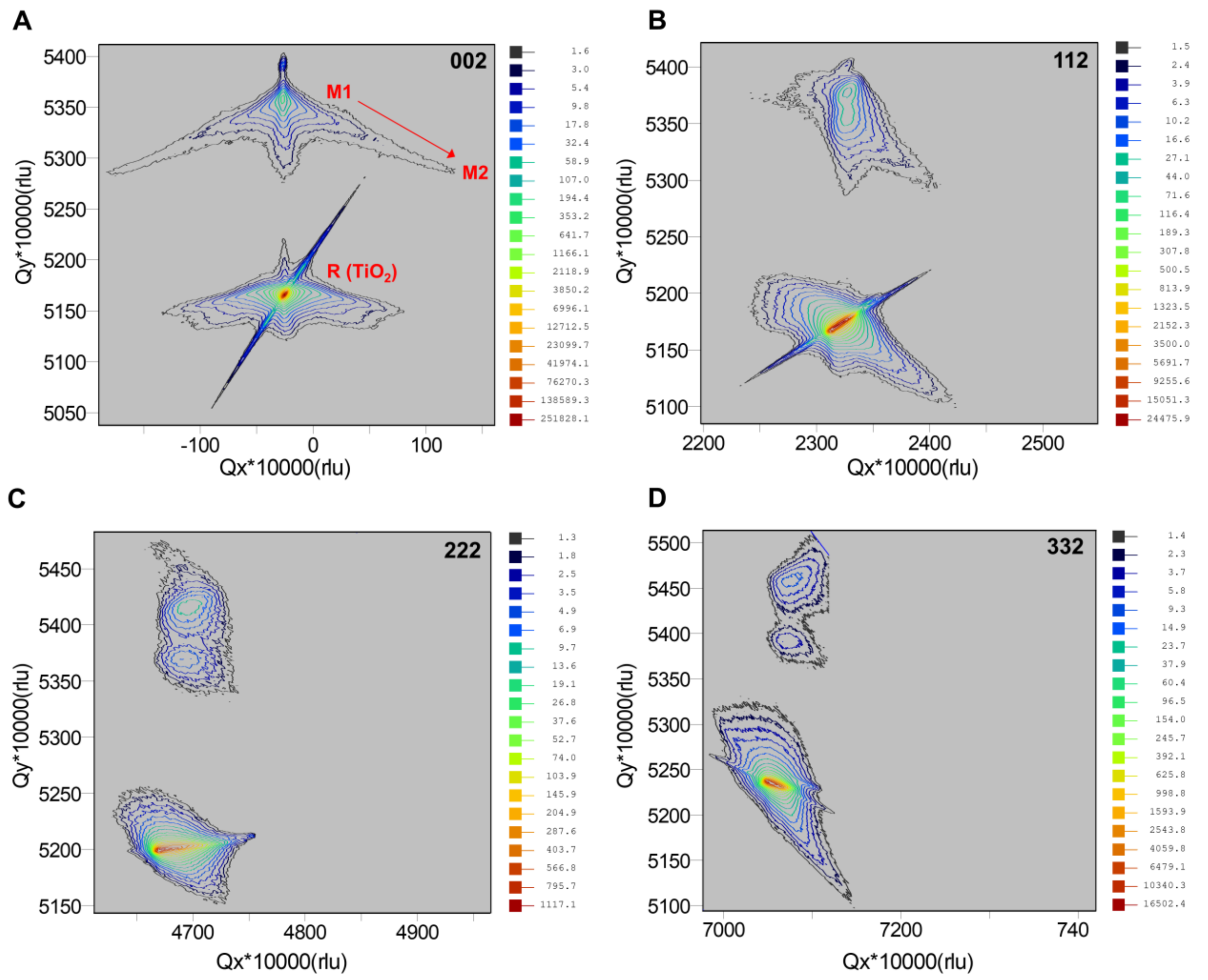

Figure S7. X-ray reciprocal space mapping (RSM) of the deposited VO_2 film along out of plane $[001]_R$ and in-plane $[110]_R$ on TiO_2 substrate: (A) the projection of $[002]_R$ demonstrating phase coexistence (M1 and M2), (B) the projection of $[112]_R$ reflection, (C) the projection of $[222]_R$ and (D) the projection of $[332]_R$ orientation.

Ellipticity and strain mapping

The ellipticity analysis was carried out using the open-source atomic resolution image analysis package Atomap [13]. Note that these analyses are performed on the real space images rather than Fast Fourier Transform (FFT) images conventionally used in geometrical phase analysis (GPA). The latter method has been shown to create artifacts and is limited to the spatial resolution of about 1 nm [14]. In our employed method, the precision (resolution) of the measurements is reached to a few picometers (e.g. 3.5 pm) order of magnitude (the pixel size of the STEM images). In this tool, the atomic positions are precisely determined in the sublattice by finding the atomic center of mass and refined by fitting 2-D gaussian distribution. Both ellipticity and strain mappings were conducted on the V-atom (cation) sublattice.

For the strain maps, the STEM image was analyzed using an in-house developed software called STEMfit[14]. The image was filtered to remove noise using a combination of truncated singular value decomposition and gaussian convolution. The filtered image was binarized using a Niblack local thresholding algorithm. The centroids of contiguous areas of above-threshold pixels in the binarized image were used as the atomic column positions for further processing. The local in-plane and out-of-plane lattice parameters were calculated for each atom by determining distances between the atom and its neighbors. The pixel size was calculated from the known lattice parameters of the TiO_2 substrate in the lower part of the image. This pixel size was used to convert the local lattice parameter values in pixel units into physical length units. Finally, the local strain was calculated as the ratio of the local lattice parameter to the bulk lattice parameter. The iDPC images used for ellipticity and strain analyses are given in Fig. S8. The scanning effect of STEM imaging might have a minor influence on the local strain values, however, the differences observed in the strain behavior of Zones I, II, III are still valid.

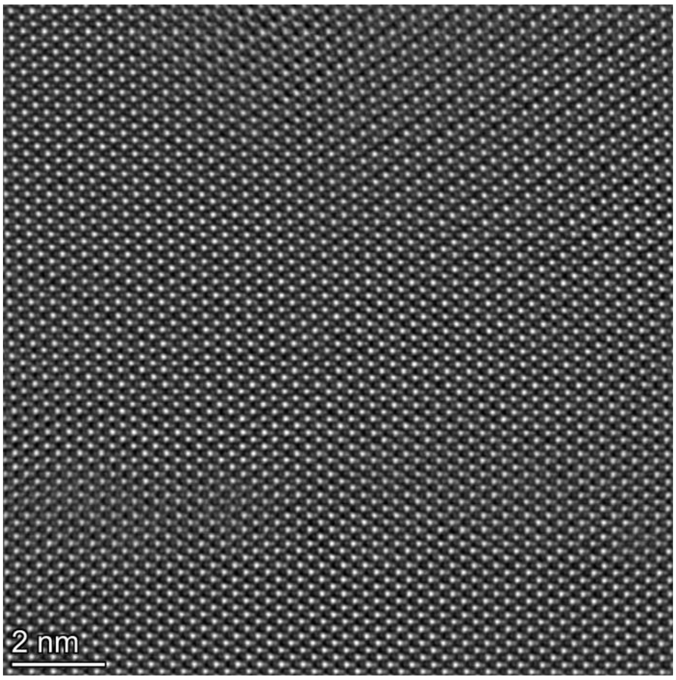

Figure S8. The iDPC image of the studied area of the film (Fig. 2C) which is used for ellipticity analysis map presented in Fig. 2D, the same image is used for strain analysis (this area includes Zones I, II and III).

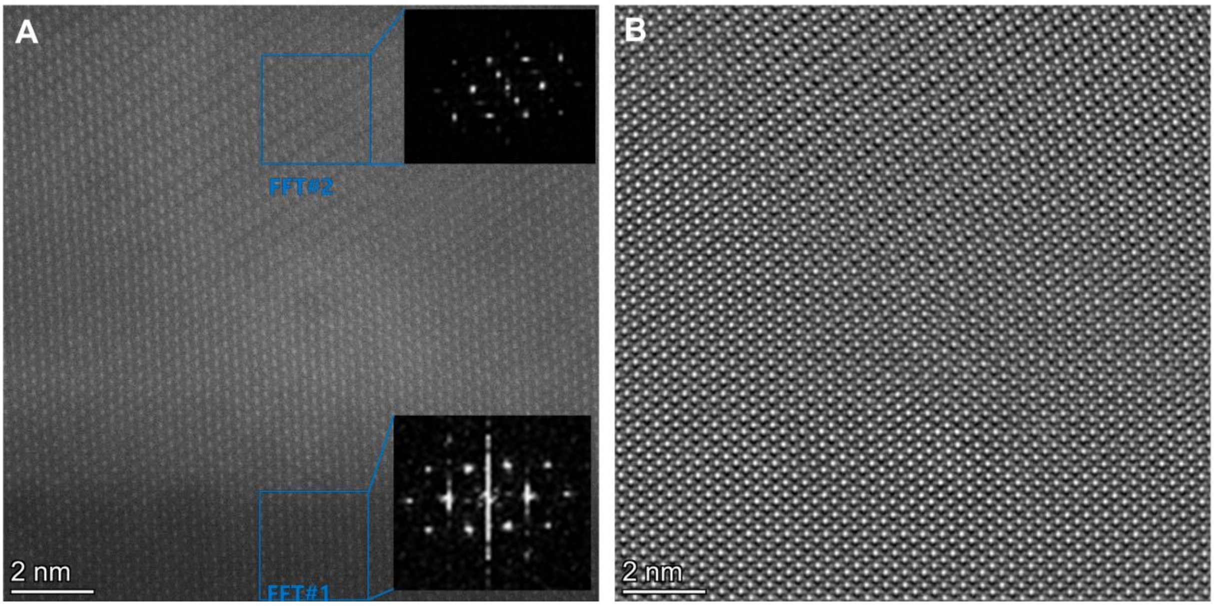

Figure S9. Extra figures for demonstrating the atomic structure of the studied cross-section of VO_2 on [001] TiO_2 substrate, (A) HAADF image together with the FFT images of TiO_2 substrate and fully dimerized M1 (A) phase, (B) corresponding iDPC image of the same region.

Electron energy loss spectroscopy (EELS) analysis

The raw data was first processed using HyperSpy Python library, with equal box regions within the layers to avoid re-normalization issues across various layers on the same sample. The core-loss regions were fitted with a power law, while no background subtraction was applied to low-loss regions for reasons discussed ahead. In Fig. S10, we have (A) ADF image of the cross-section near the interface with a selected area (shown with dotted green box) used to take EELS spectra. (B) shows the spectra (without any vertical offsets) near low-loss region with the zero loss peak cropped to show the plasmon and the $M_{2,3}$ edges with better clarity.

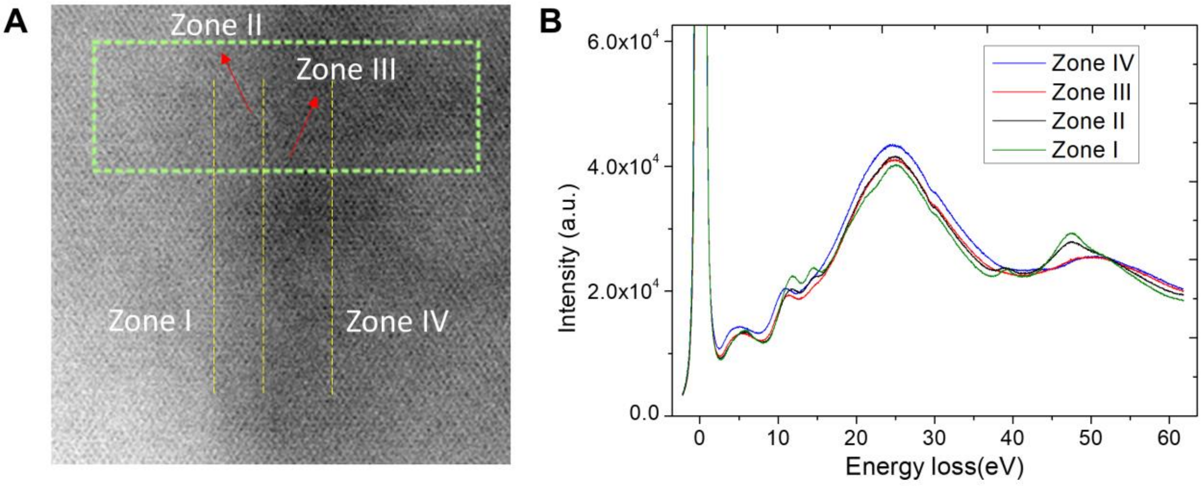

Figure S10. (A) ADF image of a cross-section of VO_2 on TiO_2 (001) with selected area for EELS, (B) EELS spectra of the low-loss region with zero-loss peak cropped to show the plasmon and $M_{2,3}$ peaks with higher resolution.

Similarly, in Fig. S11 we show DF image of the cross-section in (A), with high-loss spectra taken from the pink box shown in (B) which encompasses the V and O core-loss regions. From (A), it is trivial to find the TiO_2 substrate and M1 regions as being the furthest from the interface and selecting a box region to integrate the spectra from. Moving from the substrate (Zone I), we chose interface region (Zone II) from the HAADF image and confirmed from spectra integration that there was an appearance of the V peak. Then, the next region M2 (Zone III) was selected where we observed another substantial increase in the V peak. We can see that this is in a good agreement to the division of the layers for direct image analysis as discussed in the paper. This strategy gives us substantial differences between the spectra for the different layers despite all of them having very similar O peak. In particular, the substrate (Zone I) has no V peak at all, while the interface (Zone II) has a very weak V peak in comparison to the

similar peaks for M2 (Zone III) and M1 (Zone IV) regions. To analyze if there was any differences in peaks as well as L2/L3 ratio between M1 and M2 layers, we applied the Pearson method[15]in which integration is done with the baseline going from background to the start of the next peak, as shown in Fig. S12. This method was chosen for its simple execution which can still give relevant information if there are changes in the L3/L2 ratio. Upon measuring this for the different layers, we see no substantial differences in the ratio beyond the error margin generated due to the poor signal-to-noise ratio.

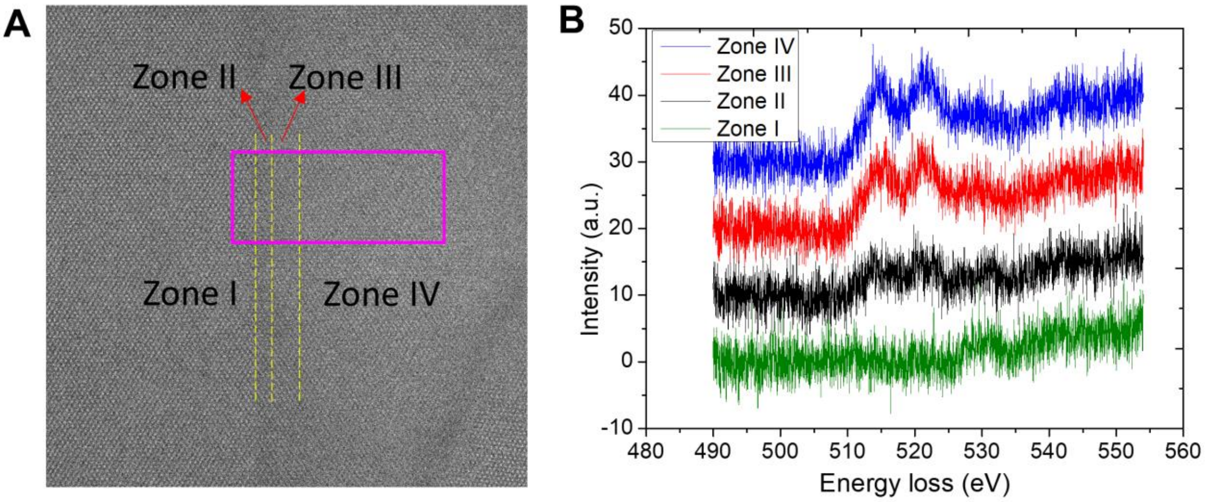

Figure S11. (A) DF image of cross-section of VO_2 on TiO_2 (001) with selected area for EELS, (B) EELS spectra of the high-loss region including V and O core-loss peaks with vertical offsets for clarity.

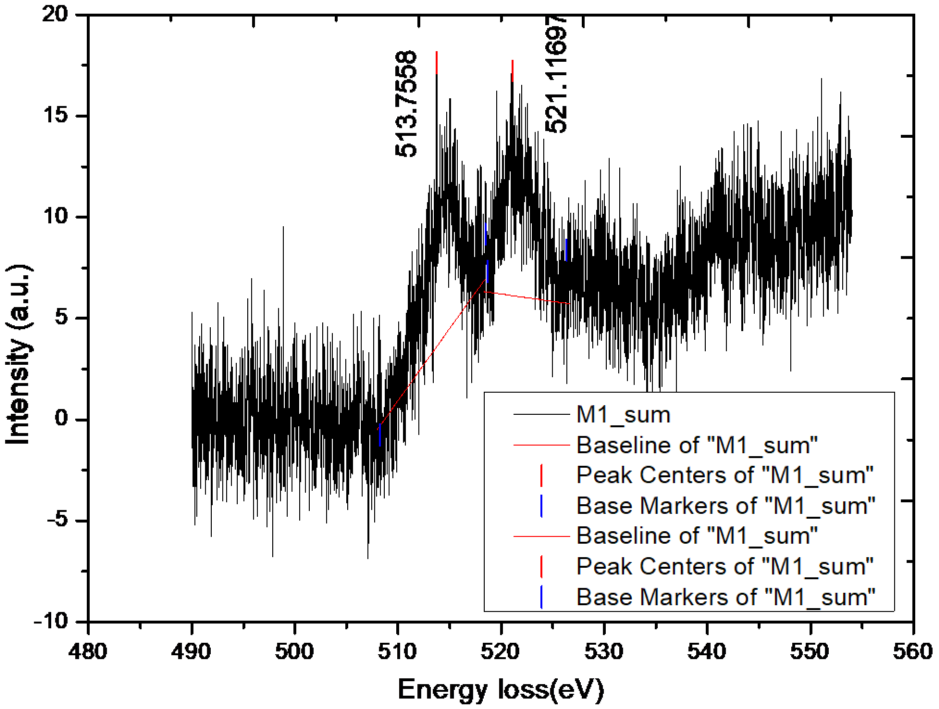

Figure S12. EELS core-loss spectra for M1 region (Zone IV) showing the V core-loss peaks as well as the baseline used for integration.

Therefore, further analysis was done using the low-loss region. The four layers in Fig. S10 (A) are again demarcated to be similar to the direct image analysis. From the low-loss region, the value of the band-gap can be determined by measuring the first peak after the zero-loss peak. Then, the linear fit to the slope of the low energy side of this peak can be extrapolated and the intercept on the energy axis is a measure of the band gap. To do this properly, first the contribution of the zero-loss peak (tail) should be subtracted properly. However, due to the orders of magnitude difference in the intensity of the zero-loss peak and the peak at 5 eV, this subtraction cannot be performed in a reliable manner and readily would lead to artifacts as shown in Fig. S13B.

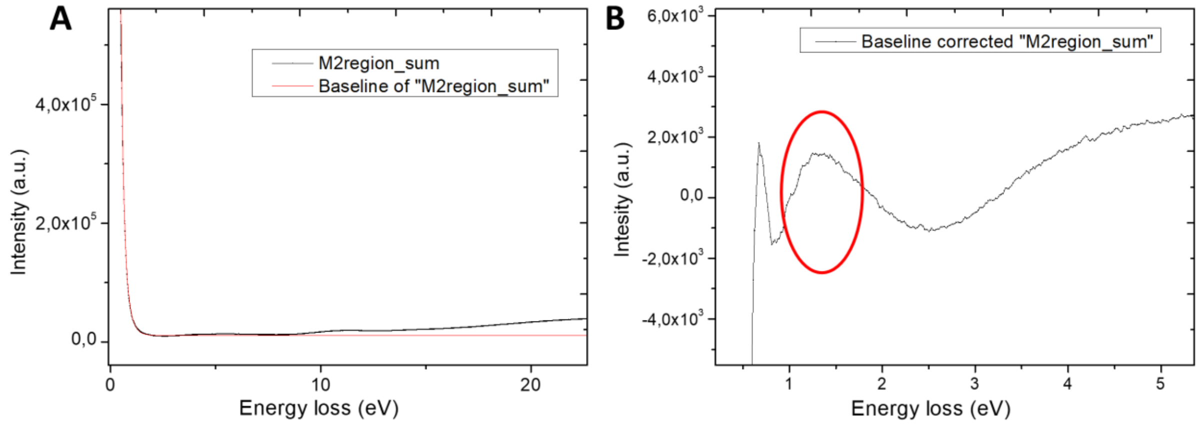

Figure S13. (A) Low-loss spectra for M2 region with the baseline created by fitting a power law onto the tail of the zero-loss peak, (B) an artifact peak generated (between 1-2 eV) before the first original peak in the spectra.

Since we wanted to have a consistent strategy to measure band-gap from the peaks, we performed an alternative approximate method for the normalization on the X- and Y-axis. All the curves were aligned to have zero-loss peak center at 0 eV energy loss to align them in X-axis, shown in Fig. S14 (A). This already gave us an indication of the differences in band-gap as can be seen in Fig. S14 (B), from the center of the first peak after zero-loss peak which can be associated with band-gap very strongly. The Y-alignment was done by making the minima after the zero-loss peak to be zero for the substrate. With the normalization done, the aforementioned extrapolation of the slope gave us the band-gaps of the different regions to be 2.89 eV for Zone I, 2.65 eV for Zone II, 2.43 for Zone III, and 1.92 for Zone IV.

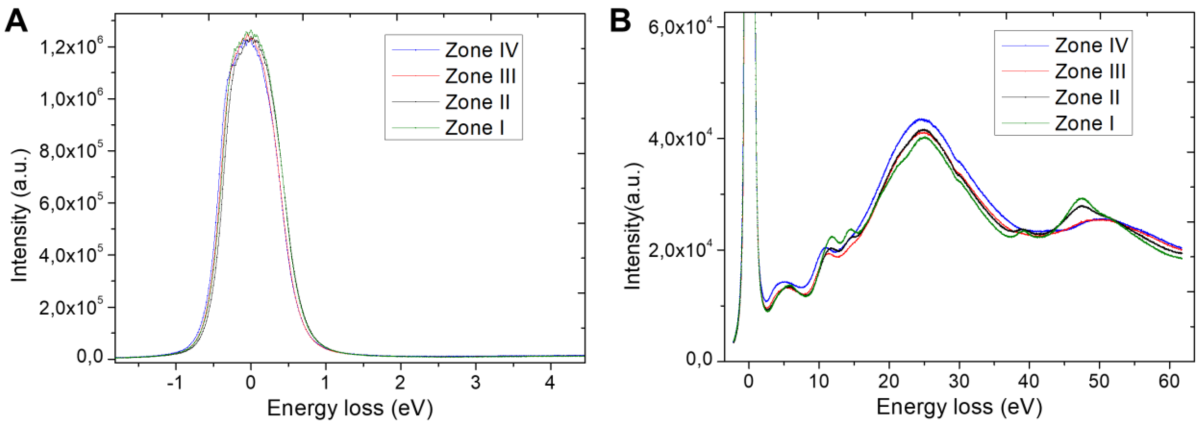

Figure S14. (A) Zero loss peak alignment has been done to be at energy loss = 0 eV, (B) the entire EELS spectra with this alignment

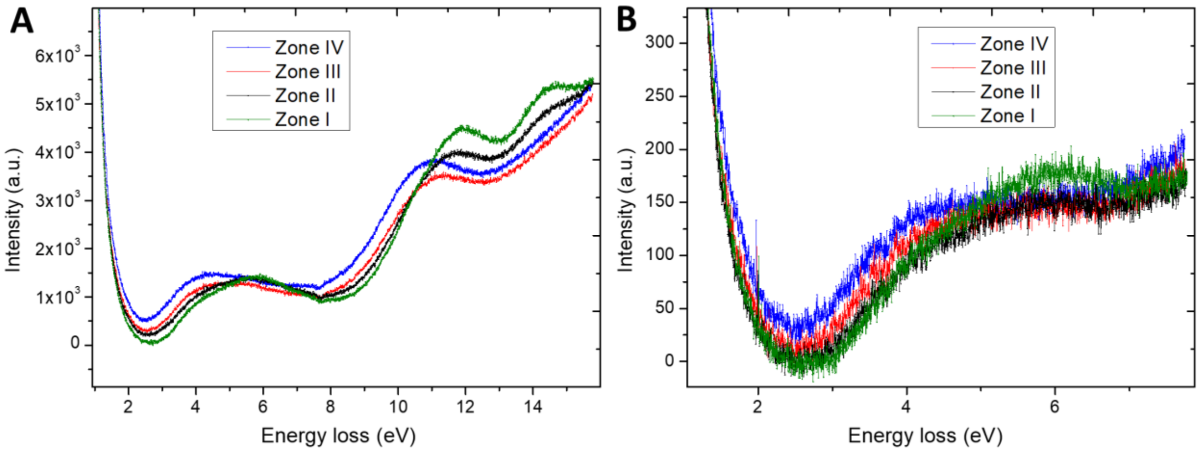

Figure S15. EELS spectra from different regions of the sample taken with the spectrometer dispersion of (A) 16 eV and (B) 8 eV.

We also took spectra from multiple regions on the sample including with different spectrometer dispersion, as shown in Fig. S15. From Fig. S15 (A), similar analysis gives us the band-gaps as 2.91 eV for Zone I, 2.51 eV for Zone II, 2.38 for Zone III, and 1.94 for Zone IV; and from Fig. S15 (B), similar analysis gives us the band-gaps as 3.03 eV for Zone I, 2.89 eV for Zone II, 2.67 for Zone III, and 2.37 for Zone IV. Although the individual band-gap values differ slightly, the differences in the band-gaps remain consistent over many sample regions. This is a great benefit from this strategy along with its ease and simplicity of execution.

References

(1) Momma, K.; Izumi, F. VESTA3 for Three-Dimensional Visualization of Crystal, Volumetric and Morphology Data. *J Appl Crystallogr* **2011**, *44* (6), 1272–1276. https://doi.org/10.1107/S0021889811038970.

(2) McWhan, D. B.; Marezio, M.; Remeika, J. P.; Dernier, P. D. X-Ray Diffraction Study of Metallic V${\mathrm{O}}_{2}$. *Phys Rev B* **1974**, *10* (2), 490–495. https://doi.org/10.1103/PhysRevB.10.490.

(3) Andersson, G.; Parck, C.; Ulfvarson, U.; Stenhagen, E.; Thorell, B. Studies on Vanadium Oxides. II. The Crystal Structure of Vanadium Dioxide. *Acta Chem Scand* **1956**, *10*, 623–628.

(4) Meagher, E. P.; Lager, G. A. Polyhedral Thermal Expansion in the TiO 2 Polymorphs; Refinement of the Crystal Structures of Rutile and Brookite at High Temperature. *Can Mineral* **1979**, *17*, 77–85.

(5) Longo, J. M.; Kierkegaard, P.; Ballhausen, C. J.; Ragnarsson, U.; Rasmussen, S. E.; Sunde, E.; Sörensen, N. A. A Refinement of the Structure of VO2. *Acta Chem Scand* **1970**, *24*, 420–426.

(6) Rafferty, B.; Nellist, D.; Pennycook, J. On the Origin of Transverse Incoherence in Z-Contrast STEM. *J Electron Microsc (Tokyo)* **2001**, *50* (3), 227–233. https://doi.org/10.1093/jmicro/50.3.227.

(7) Lazić, I.; Bosch, E. G. T.; Lazar, S. Phase Contrast STEM for Thin Samples: Integrated Differential Phase Contrast. *Ultramicroscopy* **2016**, *160*, 265–280. https://doi.org/10.1016/j.ultramic.2015.10.011.

(8) de Graaf, S.; Momand, J.; Mitterbauer, C.; Lazar, S.; Kooi, B. J. Resolving Hydrogen Atoms at Metal-Metal Hydride Interfaces. *Sci Adv* **2020**, *6* (5), eaay4312. https://doi.org/10.1126/sciadv.aay4312.

(9) Nukala, P.; Ahmadi, M.; Wei, Y.; de Graaf, S.; Stylianidis, E.; Chakrabortty, T.; Matzen, S.; Zandbergen, H. W.; Björling, A.; Mannix, D.; Carbone, D.; Kooi, B.; Noheda, B. Reversible Oxygen Migration and Phase Transitions in Hafnia-Based Ferroelectric Devices. *Science* **2021**, *372* (6542), 630–635. https://doi.org/10.1126/science.abf3789.

(10) Barthel, J. Dr. Probe: A Software for High-Resolution STEM Image Simulation. *Ultramicroscopy* **2018**, *193*, 1–11. https://doi.org/https://doi.org/10.1016/j.ultramic.2018.06.003.

(11) Marini, C.; Pascarelli, S.; Mathon, O.; Joseph, B.; Malavasi, L.; Postorino, P. Tracking Competitive Lattice Distortions in Strongly Correlated VO2-Based Systems: A Temperature-Dependent EXAFS Study. *Europhys Lett* **2013**, *102* (6), 66004. https://doi.org/10.1209/0295-5075/102/66004.

(12) Budai, J. D.; Hong, J.; Manley, M. E.; Specht, E. D.; Li, C. W.; Tischler, J. Z.; Abernathy, D. L.; Said, A. H.; Leu, B. M.; Boatner, L. A.; McQueeney, R. J.; Delaire, O. Metallization of Vanadium Dioxide Driven by Large Phonon Entropy. *Nature* **2014**, *515* (7528), 535–539. https://doi.org/10.1038/nature13865.

(13) Nord, M.; Vullum, P. E.; MacLaren, I.; Tybell, T.; Holmestad, R. Atomap: A New Software Tool for the Automated Analysis of Atomic Resolution Images Using Two-Dimensional Gaussian Fitting. *Adv Struct Chem Imaging* **2017**, *3* (1), 9. https://doi.org/10.1186/s40679-017-0042-5.

(14) van der Veer, E. STEMfit Limited Functionality 2023-06-15. Zenodo June 2023. https://doi.org/10.5281/zenodo.8042185.

(15) Pearson, D. H.; Fultz, B.; Ahn, C. C. Measurements of 3d State Occupancy in Transition Metals Using Electron Energy Loss Spectrometry. *Appl Phys Lett* **1988**, *53* (15), 1405–1407. https://doi.org/10.1063/1.100457.
